# Synthetic Mechanisms in the Formation of SnTe Nanocrystals

**DOI:** 10.1021/jacs.1c11697

**Published:** 2022-03-29

**Authors:** Sean W. O’Neill, Todd D. Krauss

**Affiliations:** † Materials Science Program, University of Rochester, 4011 Wegmans Hall, Rochester, New York 14627, United States; ‡ Department of Chemistry, University of Rochester, 404 Hutchison Hall, Rochester, New York 14627, United States; ¶ Institute of Optics, University of Rochester, 480 Intercampus Drive, Rochester, New York 14627, United States

## Abstract

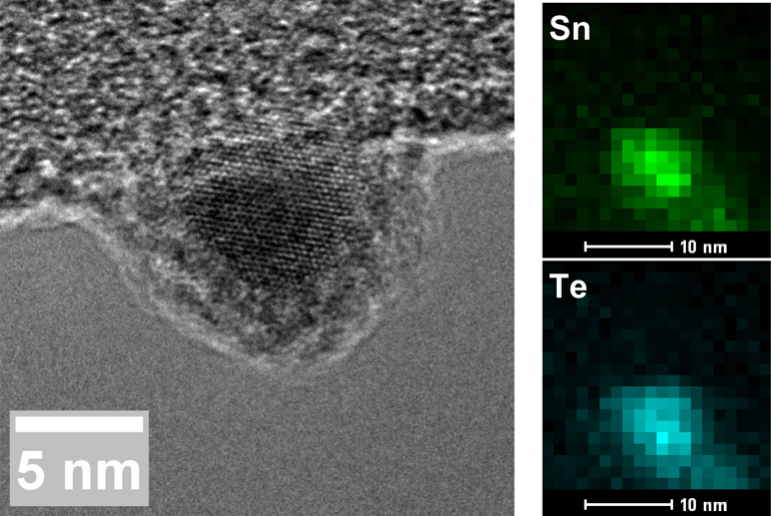

Infrared active colloidal semiconducting nanocrystals (NCs) are important for applications including photodetectors and photovoltaics. While much research has been conducted on nanocrystalline materials such as the Pb and Hg chalcogenides, less toxic alternatives such as SnTe have been far less explored. Previous synthetic work on SnTe NCs have characterized photophysical properties of the nanoparticles. This study focuses on understanding the fundamental chemical mechanisms involved in SnTe NC formation, with the aim to improve synthetic outcomes. The solvent oleylamine, common to all SnTe syntheses, is found to form a highly reactive, heteroleptic Sn-oleylamine precursor that is the primary molecular Sn species initiating NC formation and growth. Further, the capping ligand oleic acid (OA) reacts with this amine to produce tin oxide (SnO_*x*_), facilitating the formation of an NC SnO_*x*_ shell. Therefore, the use of OA during synthesis is counterproductive to the formation of stoichiometric SnTe nanoparticles. The knowledge of chemical reaction mechanisms creates a foundation for the production of high-quality, unoxidized, and stoichiometric SnTe NCs.

## 1 Introduction

Interest in novel infrared (IR) active materials is important for various applications including IR photodetectors, lasing, and light sources (e.g., LEDs).^1−4^ IR-active colloidal semiconductor nanocrystals (NCs) have emerged as promising candidates for such applications because of the tunability of their optical properties and solution processability.^5−7^ The best studied IR-active NC materials are the metal chalcogenides PbE and HgE (E = S, Se, Te), although limited investigation has also been conducted on other materials including III–V semiconductors such as InSb and plasmonic materials such as doped semiconductors and metal oxides.^8^

Another binary semiconductor material, SnTe, presents a less toxic alternative to PbE and HgE within the short-wave and mid-wave infrared regions.^9^ In addition to its reduced toxicity, bulk SnTe has several novel qualities not typically found in other IR NCs, including its inverted direct band gap, self-doped nature caused by Sn vacancies, as well as its characterization as a topological crystalline insulator.^10−12^ Although these unique properties make SnTe interesting to study for IR applications, its nanoscale photophysical properties are poorly understood. For example, SnTe NCs exhibit a size-tunable absorption peak ranging from 1.5 to greater than 3 μm.^13^ An initial hypothesis proposed that this feature was excitonic in nature,^13^ but other studies have suggested that it could arise from localized surface plasmon resonance^11^ or the formation of dopant states (i.e., absorption into an intra-band gap state arising from nonstoichiometry).^14,15^ These alternative explanations have been proposed on account of the feature’s exceptional broad line width, which would be unusual for an excitonic feature from a direct band gap semiconductor. Work from Vaxenburg et al. suggests that this peak is an excitonic transition shifted by the Burstein–Moss effect, which is a phenomenon whereby the apparent optical band gap of a semiconductor is blue-shifted on account of excess charge carriers being located in band edge states due to doping. The excess charge carriers responsible for the shift are attributed to material nonstoichiometry.^12^ This lack of understanding demonstrates that further investigation of the synthesis, structure, and properties of SnTe NCs is warranted.

Complicating the use of SnTe is its susceptibility to oxidation on exposure to air, which has been observed to be rapid and substantive both in the bulk and at the nanoscale.^11,14−17^ Bulk SnTe oxidation forms Sn(IV), Te(0), and Te(IV) species, identified largely through the formation of SnO_2_, elemental Te, and Te suboxide (TeO_*x*_, 0 < *x* < 2) alongside lesser amounts of TeO_2_ and SnTeO_*x*_.^16^ Additional studies confirmed the greater propensity of Sn to oxidize with respect to Te.^17^ Interestingly, despite the rapid oxidation of SnTe, current work on SnTe NCs has reported a range of Sn:Te ratios covering approximately 1:1.1 to 1.2:1 as measured by energy dispersive X-ray spectroscopy (EDS).^11,13,14^ Such stoichiometric or near-stoichiometric NC compositions suggest a lack of SnO_*x*_ formation; however, an amorphous tin-rich surface oxide is known to form.^11,15^ In contrast, an investigation of tin chalcogenide oxidation using ^119*m*^Sn Mössbauer spectroscopy reported SnTe NCs having a Sn(IV):Sn(II) ratio of 1.2:1 (i.e., 55:45%).^18^ While quantitatively in agreement with bulk studies using XPS,^16,17^ if we estimate the overall NC composition as being derived from Sn^4+^O_2_ and Sn^2+^Te, these results imply a Sn:Te composition of 2.2:1, which is inconsistent with compositions measured via EDS. Because of the limitations of EDS, it can be difficult to determine if an NC sample is stoichiometric with a homogeneous distribution of Sn and Te or presents as approximately stoichiometric because the Sn-deficient core is compensated by a Sn-rich oxide shell. This may potentially explain this apparent inconsistency.

Previous synthetic work on SnTe NCs have characterized some photophysical properties of the nanoparticles, but a targeted investigation of the chemical formation mechanism for SnTe has not been attempted. This work details our investigation of the unique chemical and structural properties of SnTe nanocrystals. SnTe NCs were synthesized with mean diameters between 7.2 ± 0.8 and 8.9 ± 1.2 nm. XPS elemental composition data show significant oxidation of the NCs despite the synthesis and workup in a completely air-free environment. To explain the oxidation, we used solution NMR spectroscopy to follow the kinetic evolution of the molecular Sn and Te precursors involved in the SnTe NC synthesis. From these data we developed a synthetic mechanism that helps to explain the unusual oxidation in this material and provides routes for oxidation mitigation. Specifically, we show that oleic acid (OA) is a source of oxygen for the formation of the consistently observed amorphous SnO_*x*_ shell. Thus, the ubiquitous use of OA as an NC capping ligand across a myriad of NC systems is in fact deleterious to the formation of phase-pure SnTe nanocrystals. Further, we find that oleylamine (OAm) and the Sn precursor bis(bis(trimethylsilyl)amino)tin (tin silylamide) form a highly reactive tin oleylamine that is the primary molecular Sn species involved in SnTe NC formation. While in general OAm is considered a coordinating solvent during NC growth and a weak surface ligand,^19−21^ it has been shown to have a more versatile role across certain Cd NC systems where it can influence precursor reactivity and final NC diameter.^22−25^ Similarly, during SnTe NC syntheses OAm is not a simple ligand or solvent, but it is an active reagent that we determined has a significant impact on final nanoparticle size. Summarily, we have found that OAm is an integral reagent for the formation of high-quality SnTe NCs, while the use of OA (and potentially other alkylcarboxylates and primary alcohols) are counterproductive to the synthesis of unoxidized SnTe nanoparticles. These results suggest a path forward for the production of high-quality, unoxidized, and stoichiometric SnTe NCs.

## 2 Results

**Figure 1:**
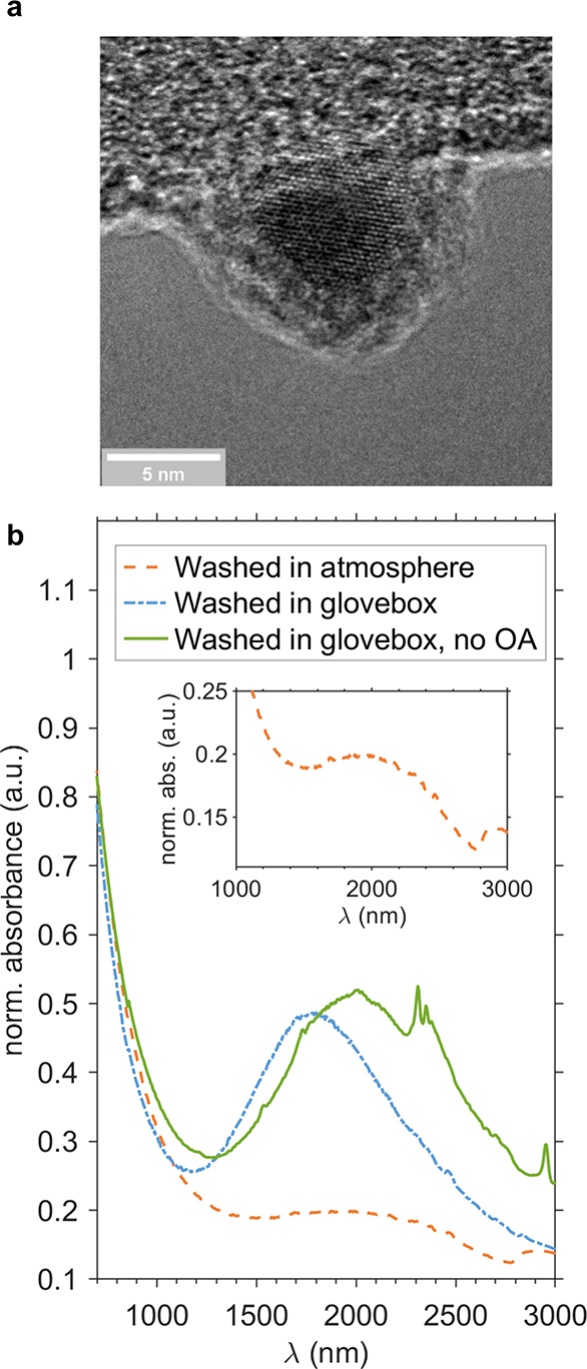
Characterization of SnTe NCs. (a) High-resolution transmission electron micrograph of a SnTe NC that was glovebox washed and OA capped, demonstrating a visible oxide shell. (b) Comparison of absorption spectra for atmosphere-washed, OA-capped; glovebox-washed, OA-capped; and glovebox-washed, OAm-capped SnTe NCs in tetrachloroethylene. The inset shows additional detail of the atmosphere-washed, OA-capped spectrum. Spectra were normalized at λ = 650 nm.

**Figure 2:**
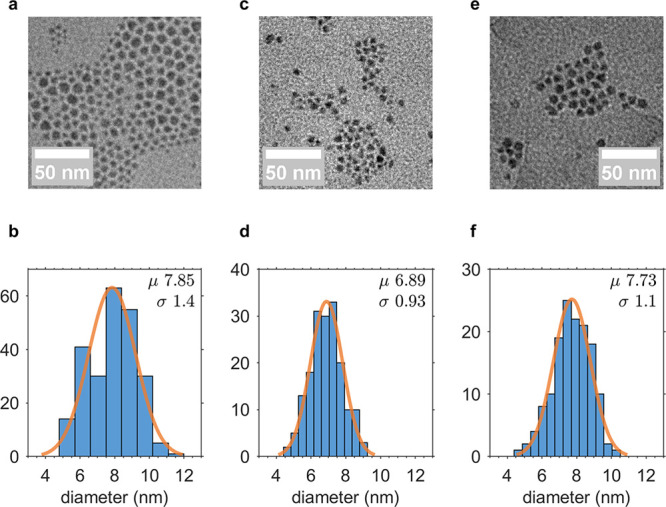
Electron microscopy characterization of SnTe NCs. Size histogram and TEM micrographs of (a, b) atmosphere-washed, OA-capped, (c, d) glovebox-washed, OA-capped, and (e, f) glovebox-washed, OAm-capped SnTe NCs.

Syntheses of SnTe NCs were conducted according to the general method of Kovalenko et al. with minor variations.^13^ In short, into a solution of dried and degassed OAm and trioctylphosphine telluride (TOPTe) was injected a solution of tin silylamide diluted in octadecene (ODE). NCs were grown for 60 s, during which time OA was injected as a capping ligand (see Supporting Information (SI) Section S1.2 for additional details). The SnTe NC synthesis was conducted at an injection temperature of 110 °C, which was among the lowest temperatures reported to produce acceptable quality NCs. This low temperature was chosen to produce small-diameter NCs that would have optical absorption resonances in the spectral range of a standard UV/visible/NIR absorption spectrophotometer, as well as to reduce the loss of low boiling point synthesis byproducts. NC purification was performed in a glovebox using dried and degassed solvents. However, regardless of the lengths taken to mitigate oxidation, samples imaged using transmission electron microscopy (TEM) consistently showed the formation of a surface oxide (Figure 1(a)). Because the samples were handled in an inert environment, we concluded that either the synthesis and/or the processing conditions were leading to this oxide formation. As noted above, SnTe NC oxidation has been reported by others, and some have suggested that the capping ligands may be a possible source of oxidation.^11,15^ To investigate this hypothesis, a single synthesis was conducted with analysis of NC products formed pre- and post-OA injection. The resulting products were purified under differing conditions, producing NCs that were (1) OA capped and purified in ambient atmosphere, (2) OA capped and purified under an inert atmosphere, and (3) OAm capped and purified under an inert atmosphere (Figures 1(b), 2(a–f), and S1). As shown in Figure 2, overall size and size distribution of SnTe NCs were generally consistent regardless of the processing conditions. Washing under atmosphere produced NCs with slightly greater polydispersity, possibly attributable to an Ostwald ripening process. Similarly, glovebox-washed NCs capped with OA had a slightly smaller mean diameter, which might be caused by etching of the NC surface during purification. In general the diameter and size distribution of our syntheses agree with previous reports.^9,12,13^ Additional details of these syntheses and purification steps may be found in the SI (Section S1.2.2).

**Figure 3:**
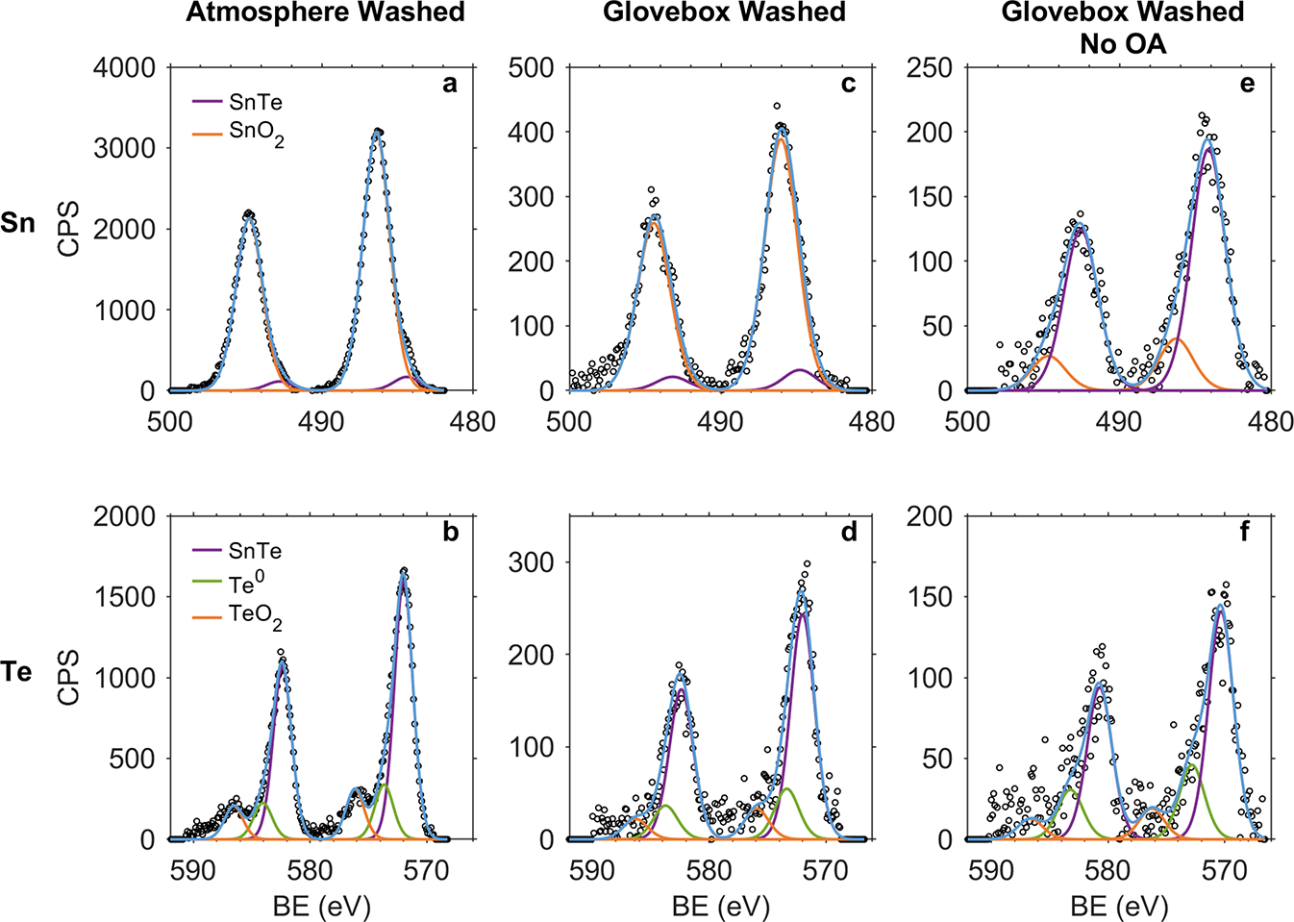
Sn and Te XPS spectra for NCs subjected to each of the three processing conditions.

Measured elemental composition is presented in Tables 1 and S1, with typical results visualized in Figures 3(a–f), S2, and S3. Peak fitting and elemental composition determination for XPS data were conducted per Section S1.14. Briefly, spectra were background corrected and fitted with pseudo-Voigt peaks (70% Lorentzian), with a minimum number of peaks employed to give a reasonable fit. Peak compound assignments (e.g., SnTe, Te^0^) were made based on measured binding energy comparison with literature values. Overall spectrum fitting (i.e., peak summation) in presented in blue in Figure 3. The integrated area of each overall fit was normalized by element-specific relative sensitivity factors, giving a measure of the composition of a given sample. In this manner sample composition was determined.

**Table 1:** Elemental [Sn]:[Te] Ratios Obtained via XPS on SnTe Nanocrystals Subject to Alternate Synthesis and Purification Conditions

	Sn:Te (mol/mol)
washed in atmosphere	2.20:1
washed in a glovebox	1.81:1
washed in a glovebox, no OA	1.24:1

All samples presented Sn-rich stoichiometries, indicating a surface enriched in Sn species, which we attribute to the formation of SnO_2_. In a related manner, the NCs presented decreasing mean Sn content with respect to Te as the samples were washed in the glovebox and OA was removed from the system. For example, the integrated area Sn:Te ratio for the atmosphere-washed, OA-capped sample (Figure 3, panels (a):(b)) is greater than that for the glovebox-washed, OAm-capped sample (panels (e):(f)). Thus, we can conclude that the presence of OA and exposure to ambient conditions each contributed in an additive fashion to increased Sn content (i.e., higher Sn:Te ratio), meaning there was increased NC oxidation through the formation of a SnO_*x*_ shell with concomitant etching of Te from the NC lattice.

### 2.1 NMR Studies of Reaction Mechanism

To understand how the NCs were becoming oxidized even while conducting all syntheses and processing under an inert atmosphere, we undertook an evaluation of the NC formation mechanisms from molecular precursors. During NC synthesis, “aliquots” or “samples” were collected for analysis by various homo- and heteronuclear NMR techniques. In summary, samples were collected during hot-injection syntheses after the addition of each precursor. Therefore, sample collections occurred: (sample 1) with the initial OAm and TOPTe mixture, (sample 2) after injection of the tin silylamide ODE mixture, (Sample 3) after injection of OA during NC growth, and (sample 4) postquenching of the reaction. Aliquots, in comparison, were collected during single-pot heat-up syntheses at (aliquot 1) 20 °C, (aliquot 2) 60 °C, (aliquot 3) 110 °C, and (aliquot 4) after OA injection and quenching. Additional details on these syntheses and NMR parameters may be found in the SI Sections S1.3 and S1.12.

**Scheme 1:**
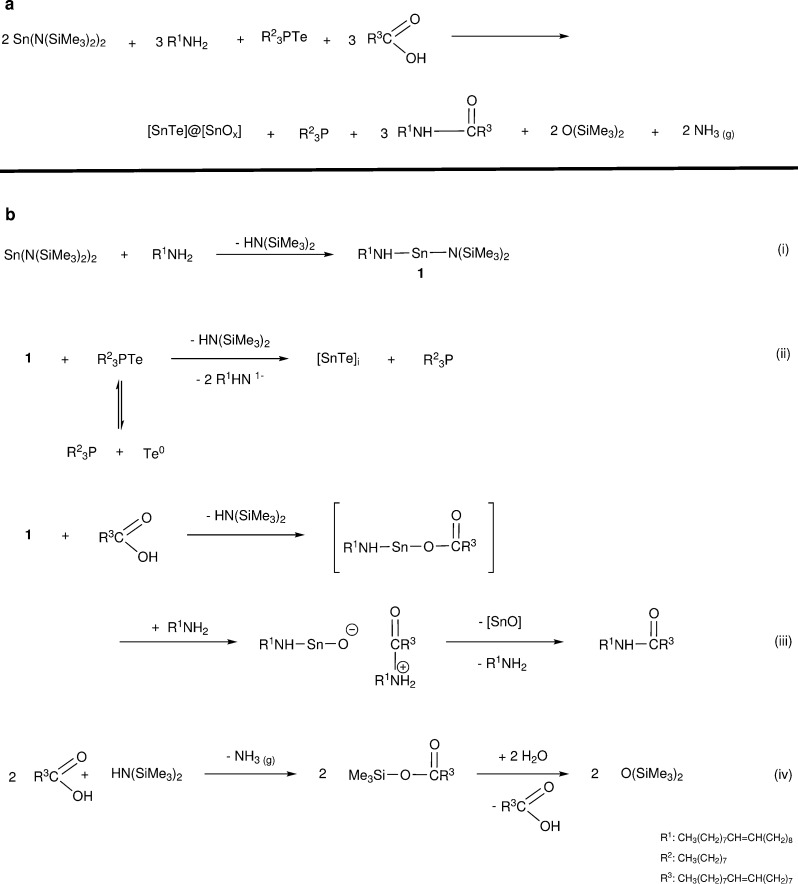
(a) Overall Formation Mechanism of SnTe NCs; (b) Constituent Formation Mechanisms Involved in SnTe Synthesis

Transformation of phosphine species, such as TOPTe, can be easily monitored by ^31^P NMR given its high sensitivity. Unlike other tertiary phosphine chalcogenides, TOPTe does not form a complex in solution but instead exists as a resonance structure between dissolved elemental Te and TOPTe (Scheme 1(b)(iii)).^19,26,27^ Because of this, rather than two distinct ^31^P peaks attributable to trioctylphosphine (TOP) and the TOP-chalcogenide, TOPTe appears as a single broadened peak whose chemical shift varies with the [Te]:[TOP] ratio in solution, with the peak found further upfield as Te content decreases. Low-temperature solution NMR can be used to resolve the TOP and TOPTe peaks, which were found at −32 and −9.3 ppm, respectively (Figure S4).^28^

**Figure 4:**
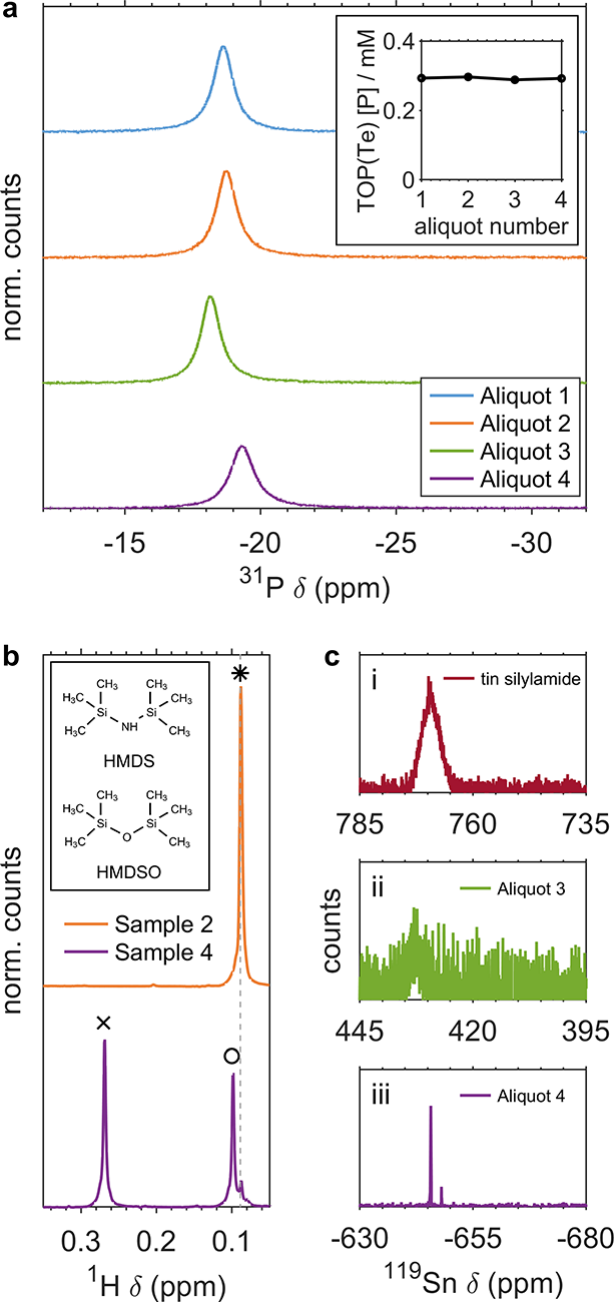
(a) ^31^P spectra of synthesis aliquot 1 through aliquot 4 displaying the TOPTe resonance alongside (inset) quantification of measured TOP + TOPTe (TOP(Te)) concentration. (b) ^1^H spectra of synthesis samples collected before (sample 2) and after (sample 4) OA injection. Identified species include (*) HMDS, (◦) HMDSO, and (×) oleate ester. The inset shows the molecular structures of HMDS and HMDSO for reference. (c) ^119^Sn spectra of (i) tin silylamide and synthesis aliquots (ii) before and (iii) after OA injection.

While many NC syntheses employing tertiary phosphines observe the formation of trioctylphosphine oxide (TOPO) during nucleation and growth,^29−32^ such is not the case for SnTe. Consistently, quantitative NMR demonstrated that greater than 95% of the P in the reaction remains as TOP or TOPTe throughout the NC synthesis. An upfield shift of the TOPTe peak during NC growth evidences a decrease in the [Te]:[TOP] ratio, as expected when Te is incorporated into SnTe NCs (Figures 4(a) and S5(k, l)). The fact that TOP does not chemically change during the course of the reaction suggests that it is simply acting as a vector for Te delivery as the NCs form. Concomitantly, ^125^Te NMR spectra indicate that unreacted Te remains as TOPTe in solution, with no observable side products formed (Figure S5(m)).

Although no other Te species were observed in solution aside from TOPTe, several molecular Sn species were formed. In early stages of syntheses, no Sn signal could be seen in ^119^Sn spectra. As NC growth progressed, we observed that a weak Sn peak began to grow in around 435 ppm, significantly upfield from the characteristic signal of tin silylamide (Figures 4(c)(i, ii) and S6). Upon OA injection, this species became unobservable and was replaced by a new peak at ^119^Sn δ ∼ −645 ppm (Figure 4(c)(iii)).

**Figure 5:**
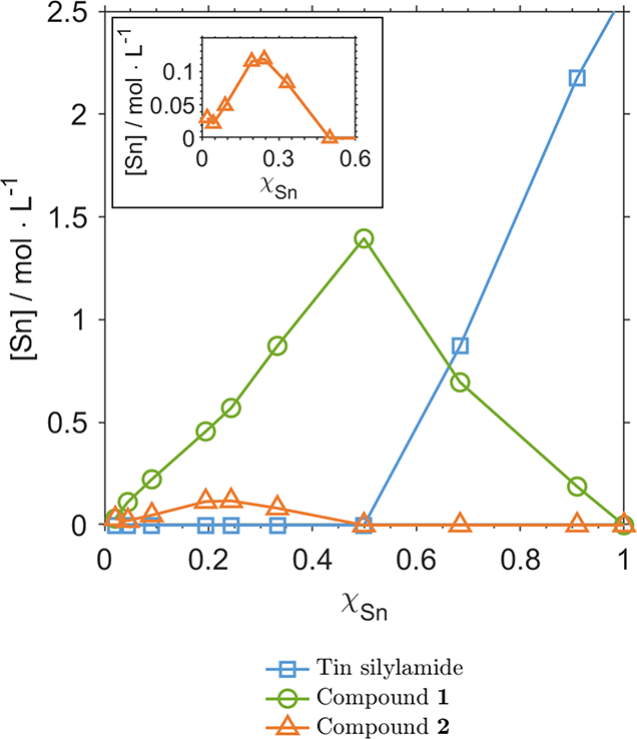
Method of continuous variation’s Job plot. The inset shows greater detail of the compound **2** region. Details on sample preparation and data analysis are provided in Sections S1.4.5 and S2.2.

The ^119^Sn peak observed during early portions of the synthesis (δ ∼435 ppm) is found significantly upfield from that of tin silylamide (δ ∼775 ppm; Figure 4(a)) and was hypothesized to arise from the reaction of OAm and tin silylamide. To better understand this molecular species, the method of continuous variation was used to construct a Job plot, which can be interpreted to determine the stoichiometry of a chemical product. Here, OA and tin silylamide were reacted under an inert atmosphere in varying ratios while maintaining a constant molar sum (Figures 5 and S7; Section S2.2).^33^ This resulted in the formation of a molecular species with a characteristic ^119^Sn chemical shift at δ ∼675 ppm. This species was not observed during NC syntheses and was taken to be a highly reactive species involved in NC formation. From the Job plot seen in Figure 5, the unknown species has a Sn:OAm ratio of 1:1, suggesting that this molecule is a heteroleptic tin amide, *N*-(octadec-9-en-1-yl)-*N*′,*N*′-bis(trimethylsilyl)stannanediamine (**1**). This same analysis was also able to reproduce the observed molecular Sn species (δ ∼435 ppm) as a minor side product, which is identified as being a product of Sn and OAm having a molecular ratio of 1:3. Several mass spectrometric techniques were employed in an attempt to further identify this molecule’s structure without success (Figure S8; Section S1.10). We speculate that it may be a tin-oleylamine molecule presenting a 1:3 Sn:OAm ratio or may result from a process utilizing three amines to yield the final side product. Although we lack a structure, this species will be referred to as **2** for simplicity (Figure 5). This molecule constitutes a minority of the Sn in the system and is not directly involved in NC formation, as evidenced by its production outside of NC syntheses.

After initial NC formation, OA is injected as a capping ligand because any earlier injection yields preferential formation of tin oleate (Sn(OA)_2_) and not SnTe NCs.^13^ The Sn species observed after OA injection (δ ∼−645 ppm) is significantly downfield from the other species encountered throughout the synthesis. It is not simply Sn oleate, however, which has a chemical shift of δ ∼−543 ppm (Figure S9). To determine the structure of this byproduct, we used ^1^H–^119^Sn and ^1^H–^13^C HMBC NMR (Figure S10) on the crude NC reaction mixture. ^1^H–^119^Sn HMBC identified several protons proximate to the Sn nuclei (Figure S10(a)). These same peaks were noted in ^1^H–^13^C HMBC spectra of the same sample to identify a Sn-proximate carbon (δ ∼178 ppm), characteristic of the OA carboxylate carbon (Figure S10(b)). These data indicate that Sn is bonding to or near the oleic acid quaternary carbon, forming an unidentified alkyl-Sn species. Unfortunately, attempts to fully isolate and characterize this molecule were unsuccessful, and analysis of synthesis aliquots by 2D NMR was too complicated by other molecular species to determine a definitive structure.

Additional mechanistic information was monitored through characteristic ^1^H and ^13^C NMR peaks, specifically those occurring in the trimethylsilyl and carboxylate regions. Monitoring of proton NMR during NC syntheses indicated the formation of hexamethyldisilazane (HMDS) from the ligands of tin silylamide. This species converted to hexamethyldisiloxane (HMDSO) upon OA injection, as well as another species identified as the silylated oleate ester (Figures 4(b) and S11). The formation of HMDSO was conclusively determined by the addition of HMDSO to a SnTe NC reaction mixture (Figure S12).

With respect to ^13^C spectra, in addition to the cabonyl signal from free OA and the Sn-containing molecule discussed above (i.e., alkyl-Sn), there is another carboxylate peak at δ ∼172 ppm. A mixture of tin silylamide, OAm, and OA was made and heated at 110 °C (see Section S1.4.1). FTIR absorption data were collected and positively identified the formation of an amide (Figure S13), indicating that tin silylamide is promoting the formation of *N*-(*cis*-9-octadecenyl)oleamide (oleyloleamide, OOA).^34−36^ Such behavior by tin silylamide has been reported previously.^37−40^ The characteristic ^13^C peak was not observed when OAm and OA were heated in the absence of tin silylamide (Figure S14). On the basis of our accumulated data (i.e., NMR, FTIR, XPS, and EDS), we propose a formation mechanism for our synthesized SnTe NCs (Scheme 1).

### 2.2 Mechanism Validation

To support our hypothesis that **1** is responsible for SnTe NC formation, we conducted a synthesis whereby a prereacted solution of tin silylamide and OAm (1 h at 110 °C) was used as the Sn precursor. Preparation of a prereacted tin silylamide–OAm precursor supplied the reaction with significantly more nuclei with respect to a typical synthesis, resulting in a greater number of NCs with a notably smaller mean diameter (∼6.5 nm down from ∼7.7 nm, Figure S15). The increased nucleation supports the conclusion that tin silylamide and OAm form a highly reactive tin alkylamine, which is the Sn species that leads to NC formation. This experiment also demonstrates that OAm is not simply a nonreactive solvent, but is an integral reagent to achieve well-formed NCs. To investigate the potential for an OAm impurity to be the reactive amine species given the 70% purity used during a typical synthesis, we conducted an NC synthesis using 98% purity OAm. The resulting NCs demonstrated a modest increase in their mean diameter to 8.6 ± 1.5 nm (Figure S16). We speculate the smaller mean diameter when using the less pure amine may be attributable to the presence of shorter chain primary amine impurities that have modestly increased reactivity compared to OAm. Further, we attempted to synthesize NCs using an equivalent volume of a tertiary amine (trioctylamine) in place of OAm to validate the role of the primary amine in NC formation. No NCs were produced during such syntheses. When using trioctylamine, the solution retained the orange color of unreacted tin silylamide upon injection and did not turn immediately black as is typically observed during NC formation. The solution remained orange for greater than 2 min, the maximum reaction time employed for SnTe NC syntheses. If the reaction was allowed to proceed, a very slow darkening was observed, with a color change to a deep brown after approximately 10 min. Attempts to purify this product using typical NC purification techniques were unsuccessful, resulting in an unstable brown precipitate that would not resuspend in any nonpolar solvent (e.g., hexanes, toluene, chloroform).

## 3 Discussion

Our investigation of the elemental composition of SnTe NCs shows that they are synthesized with a Sn-rich surface, with a greater degree of Te deficiency upon exposure to ambient atmosphere and/or OA. Even when maintained air-free throughout synthesis and processing, we have found that SnTe NCs still demonstrate notable oxidation. This air-free oxidation suggests that the use of OA during NC synthesis contributes to the formation of a surface oxide, specifically when using the synthesis described in Kovalenko et al. and potentially for SnTe NCs in general.^13^

These data indicate that the bis(trimethylsilyl)amino ligands liberated from the tin silylamide function as Brønsted bases, deprotonating OAm and forming highly reactive heteroleptic tin oleylamine **1** alongside HMDS (Scheme 1(b)(i)). Molecule **1** reacts with Te delivered by TOPTe to initiate NC nucleation and growth (Scheme 1(b)(ii)). Similar nanostructure formation via a reactive metal oleylamine species has been reported for both metal NCs (e.g., Sn, Cu, Zn, Bi) and tertiary Fe_2_GeS_4_ semiconducting nanostars, most notably using metal halides and OAm alongside hexamethyldisilazane lithium as the Brønsted base.^41−44^ Further, we identified **2** (^119^Sn δ ∼435 ppm) as a minor side product that must be produced from the interaction between OAm and tin (Figure 4(c)(ii)). Another molecular Sn species was also identified from the reaction of tin silylamide and OAm, but was not observed during NC synthesis (Figure S7), indicating that **1** is the primary reactive Sn species involved in NC formation.

### 3.1 Role of Phosphine Telluride

After Te consumption during NC formation, TOP remains in solution, while excess Te remains unreacted as TOPTe (Scheme 1(b)(ii)). This behavior is highly irregular among II–VI and IV–VI NC syntheses involving OA and TOP, which typically yield phosphine oxide and an anhydride as byproducts. Among the more common binary semiconductor syntheses (e.g., ME; M = Cd, Pb, Zn; E = S, Se, Te) chalcogens are reportedly cleaved from phosphine chalcogenides during NC formation via an acid/base mechanism where the chalcogen is transferred in the −2 oxidation state (E^2–^). The phosphine chalcogenide is subjected to nucleophilic attack by an oxygen nucleophile (e.g., free carboxylate or phosphonate ion), leading to cleavage of the chalcogen as E^2–^ alongside production of the commensurate phosphine oxide. By this mechanism, the reduction of the metal’s oleate ligand (M(O(O=C)R)_2_) and concomitant oxidation of the phosphine (R_3_PE) produces the desired binary semiconductor ([ME]_i_), phosphine oxide (R_3_PO), and oleic anhydride ((R(C=O))_2_O).^19,29−32,45^

In the context of SnTe NC synthesis, it seems the unusual phosphine reaction mechanism encountered here arises from the characteristics of the metal–ligand system. During the later phase of SnTe NC formation, when alkylcarboxylates are available, the chemical interactions of the carboxylates are dominated by reactions with silicon-containing moieties, specifically, the exchange of a trimethylsilylamino group with an oleate to ultimately form OOA (Scheme 1(b)(iii)) and the silylation of OA to produce silylated oleate ester (Scheme 1(b)(iv)). Therefore, we conclude that the lack of TOPO formation does not reflect on the nature of the phosphine–chalcogen cleavage reaction, which we believe is attributable to an acid/base interaction, but rather the dominant acid/base interaction of OA with the strongly basic trimethylsilylamino-containing ligands over TOP.

Regardless, in order for NC formation to proceed, an oxidation must occur alongside either the reduction of Te^0^ to Te^2–^ and subsequent reaction with Sn^2+^ or reduction of Sn^2+^ to Sn^0^ and reaction with Te^0^. While we cannot eliminate the possible formation of Sn^0^ via thermal decomposition and subsequent oxidation by TOPTe to form [SnTe]_i_ and TOP, this is not a likely reaction mechanism given the observed formation of **1** and the low reaction temperature employed. Alkylamines (and OAm specifically) have been reported to be mild reductants in various NC syntheses. This has been shown for the reduction of S_8_, typically to reactive H_2_S, as well as the reduction of metals for metal NC syntheses.^20,46,47^ Alternatively, limited reports of silylamino ligand oxidation may provide another explanation.^48,49^ In such work, the reaction of tin silylamide with an organodichalcogen molecule containing an amino side arm (e.g., (RE)_2_, E = S, Se) was shown to produce heteroleptic organochalcogenolato tin silylamides. Chalcogen reduction to the E^2–^ oxidation state was attributed to amine ligand oxidation via hexamethyldisilylhydrazine formation. Interestingly, coordination of the amino side arm to the metal center stabilized the molecule toward the low oxidation state of the metal via a tetrel interaction between the N free electron pair and metal center. One can envision that perhaps a similar interaction could occur between tin silylamide and the large OAm excess during SnTe NC synthesis. Unfortunately, at this time we can only speculate on the nature of this redox reaction, but it is the subject of continued inquiry.

### 3.2 Silylamine Sideproducts

While the final trimethylsilylated product during SnTe synthesis is HMDSO, the exact mechanism for its formation remains unclear. HMDS readily reacts with OA to form oleate ester and ammonia, which is evidenced by the visible evolution of gas from the solution. A comparison of the reaction of tin silylamide, OAm, and OA shows that when reacted stoichiometrically, HMDS results. However, under the same reaction conditions with an excess of OA, HMDS forms HMDSO via the oleate ester intermediate (Figure S11). Conceivably, self-condensation of oleate ester to form water and an oleic anhydride could occur, but the lack of a characteristic anhydride double peak in infrared absorption spectra (∼1730–1780, 1780–1860 cm^–1^) shows this is not the reaction pathway (Figure S13). We believe that formation occurs via reaction of oleate ester with residual water associated with dried OA. Although dried under vacuum and stored over molecular sieves, the carboxylic acid moiety has a high water affinity and some small amount may remain to react with the unstable oleate ester to form HMDSO (Scheme 1(b)(iv)).

### 3.3 Tin Silylamide

As noted previously, aliquots collected during early portions of NC syntheses do not show any observable ^119^Sn signal. The reason for this phenomenon remains unclear. We hypothesized that perhaps a resonance structure exists between NC precursors. However, a stoichiometrically equal mixture of OAm, tin silylamide, and TOPTe in deuterated THF (combined at room temperature, unheated; 150 mM Sn) showed no Sn NMR signal at any temperature between ±30 °C, suggesting resonance broadening of the NMR signal is not the cause. Alternatively, a Sn-containing molecular cluster species might explain these data, particularly given divalent tin amides’ propensity to form dimers, cubanes, and larger molecular structures.^50,51^ A cluster could be too large to resolve using solution NMR, particularly if the Sn signal is further broadened by bonding with quadrupolar ^14^N nuclei.

The exact mechanism of formation for the alkyl-Sn species is unclear. There is a body of literature regarding carbonyl activation using tin reagents, and specifically tin silylamide, whereby tin causes the carbonyl carbon to become sufficiently electrophilic that it may undergo nucleophilic attack by, for example, an amine.^37−40,52−54^ This mechanism may be generalized to other Lewis acid metal catalysts such as Pd, Ni, or Zn.^55,56^ In light of this precedent, it is most likely that the Sn nucleus is bound to one or more oleate ligands through the carboxylate moieties. This conclusion is directly supported by HMBC analysis, which demonstrates that the Sn nucleus is found in the vicinity of the carbonyl functional group. We note, however, that this alkyl-Sn was only found when all NC precursors were reacted (i.e., tin silylamide, OAm, TOPTe, and OA), suggesting that Te or TOP may be involved in its formation. The chemical shifts of Sn species cover a characteristically broad region, and therefore while this unknown Sn species may be another bi- or tetracoordinated Sn(II) molecule, it is also consistent with an oxidized tetra- or hexacoordinated Sn(IV) species.^57^

In addition to an alkyl-Sn byproduct, FTIR spectra of the synthesis aliquot indicate amide formation (Figure S14). Identification of this amide as OOA was confirmed via NMR spectra of a reacted mixture of 1:1:1 OAm:tin silylamide:OA (see Section S1.4.1 for details on sample preparation), demonstrating characteristic ^13^C and ^1^H peaks at δ ∼172 and 3.2 ppm, attributed to the quaternary carbonyl and the N-adjacent methylene H, respectively.^34^ This same carboxamide species has been reported to form in lead halide perovskite syntheses, albeit at much higher temperatures then those encountered here.^35,36^ The occurrence of the amide at the low temperatures experienced here is attributed to the presence of tin silylamide, which has been reported to facilitate the reaction of carboxylates and amines to form carboxamides and tin oxide (SnO).^37^ This also explains the notable oxidation of NCs even when maintained completely air free throughout synthesis and processing (Scheme 1(b)(iii)). Therefore, OA is functioning as an oxygen source for the formation of SnO_*x*_, which behaves as a Sn- and O-rich monomer during NC growth, facilitating the formation of the commonly observed SnO_2_ shell.

An alternative explanation is that it is HMDS (not necessarily Sn) that is promoting the formation of OOA because the formation of amides from carboxylates and primary amines using HMDS has been reported elsewhere.^58−60^ By such a mechanism, HMDS silylates the carboxylate to form a trimethylsilylated oleate ester and ammonia gas. This ester then reacts with a primary amine to form the appropriate carboxamide alongside trimethylsilanol. Trimethylsilanol can dimerize when heated, forming HMDSO and water.^61^ To investigate this mechanism, we reacted HMDS, OA, and OAm (stoichiometric ratio) at 110 °C. However, this reaction did not result in amide production, instead forming the oleate ester and unreacted OAm (Figure S14). This indicates that Sn is necessary for the amide formation reaction to proceed at the low temperatures used for SnTe synthesis and provides further evidence for the production of SnO alongside OOA.

## 4 Conclusions

In summary, we have shown that SnTe NC quality is extremely sensitive to both synthesis and postprocessing parameters, including ligand choice and degree of air exposure (Table 1; Figures S2 and S3). Our mechanistic work demonstrated that OAm is a reagent during SnTe synthesis, not just a solvent (Figures 5 and S15), and the resulting tin oleylamine 1 is necessary for the formation of NCs with well-controlled size and a reasonable size distribution. Further, we have shown that tin silylamide facilitates a reaction between OAm and OA even at low temperatures, producing SnO, which is a major source of the oxide shell encountered on these NCs (Scheme 1).

Our investigation has identified OA as a major source of oxidation during SnTe NC formation. Its subsequent elimination from the synthetic method produced NCs with reduced surface oxide formation, yielding a more stoichiometric product. Similar detrimental effects of ligands may be active in other NC systems, particularly those employing carboxylates or alcohols, and our work suggests additional scrutiny should be afforded to such potential undesirable interactions. Conversely, we also demonstrate previously unidentified desirable interactions, contributing to the growing body of NC systems whereby OAm is an unintended reagent yielding the formation of reactive metal amines that facilitate NC formation. Lastly, the absolute necessity to purify and handle SnTe in an air-free environment is paramount, as this work shows. While we have achieved significant reduction in oxide formation during NC synthesis and purification, additional work remains necessary to increase the stability of SnTe under atmospheric conditions and to produce stoichiometric NCs.

## Supporting Information Available

SnTe NC synthesis protocols, infrared absorption spectra, TEM and sizing histograms, characterization by XPS and discussion of its use as a surface characterization technique, 1D and 2D NMR spectra, discussion of the method of continuous variation, and mass spectrometry data (PDF)
