# Supplementary material for: Synthetic Mechanisms in the Formation of SnTe Nanocrystals
Source: J Am Chem Soc. 2022 Mar 29;144(14):6251–60. doi: 10.1021/jacs.1c11697 (PMC9011400; doi:10.1021/jacs.1c11697)
Supplement: Supplementary file 1 — ja1c11697_si_001.pdf [file ja1c11697_si_001.pdf]

Synthetic Mechanisms in the Formation of SnTe Nanocrystals

Sean W. O'Neill[†] and Todd D. Krauss[*, ‡, ¶, †]

[†] *Materials Science Program, University of Rochester, Rochester, NY*

[‡] *Department of Chemistry, University of Rochester, Rochester, NY*

[¶] *Institute of Optics, University of Rochester, Rochester, NY*

[*]Email: todd.krauss@rochester.edu

S1 Experimental Design

S1.1 Chemicals and Materials

Chemicals used: Acetonitrile (MeCN, 99.5%, Fisher Scientific), Benzene-d6 (C_6D_6, 99.5% D, Cambridge Isotope), Bis(bis(trimethylsilyl)amino tin(II) (tin silylamide, Sigma), Chloroform ($CHCl_3$, anhydrous, $>$ 99%, Sigma), Diphenylditelluride (Ph_2Te_2, 98%, Sigma), Hexamethyldisilazane (HMDS, 99%, Sigma) Hexamethyldisiloxane (HMDSO, 98.5%, Sigma), Hexamethylphosphoramide (HMPA, 99%, Sigma) Octadecene (ODE, 90%, Sigma), Oleic acid (OA, 90%, Sigma) Oleylamine (OAm, 70%, Sigma), Oleylamine (OAm, 98%, Sigma), Tellurium (Te, 99.999%, Sigma), Tetrachloroethylene (TCE, anhydrous, >99.0%, Sigma), Tetrahydrofuran-d8 (THF-d8, 99.5% D, Cambridge Isotope), Tetramethyltin (Me_4Sn, 99%, Sigma), Tin oleate (85%, Gelest), Toluene (99.5%, Fisher Scientific), Toluene-d8 (99.5% D, Cambridge Isotope), Trioctylamine (TOA, 97%, TCI), Trioctylphos-

phine (TOP, 97%, Sigma), and Trioctylphosphine oxide (TOPO, 99%, Sigma, ReagentPlus). Toluene and MeCN were dried and deoxygenated on a Glass Contour System (Pure Process Technology, LLC) and stored over 3 Å molecular sieves in an N_2 glovebox. 3 Å molecular sieves (EMD Millipore) were activated by heating under vacuum on a Schlenk line at 250 °C for 12 hours, and then at room temperature for an additional 6 hours.

S1.2 SnTe NC Synthesis

All materials were dried and degassed prior to storage in an N_2 glovebox unless received in a state such that this was unnecessary (i.e., packed under inert atmosphere because the material is pyrophoric, hydrolytic, hygroscopic, etc.). Solvents were stored over 3 Å molecular sieves (including those purchased as anhydrous) and filtered using 0.22 µm PTFE syringe filters before use. All reactions were conducted under a nitrogen atmosphere. Reactions occurred in three-neck round bottom flasks, equipped with water-cooled condensers, under positive nitrogen pressure unless otherwise indicated. 0.7 M TOPTe (1:3 Te:TOP mol/mol) was prepared by combining Te pellet with an appropriate volume of TOP and either stirring overnight at 60 °C or stirring for two hours at 200 °C. The final TOPTe solution was allowed to cool to room temperature prior to use.

NC synthesis was a slightly modified technique from Kovalenko et al.,[1] and began by combining 14 mL OAm (70%) in a 100 mL three-neck round bottom flask and degassing at 120 °C for at least one hour to remove oxygen and water. After degassing, this flask was placed under N_2 and 1 mL TOPTe (0.7 mmol Te) was injected. The temperature of the flask was adjusted depending on the desired final NC diameter (110 - 180 °C, with higher temperatures yielding larger particles). After temperature stabilization, 6 mL of 67.1 mM tin silylamide in ODE (0.4 mmol Sn) was rapidly injected, yielding an immediate color change from orange to black. After a predetermined period of growth (60 – 120 s), the reaction was quenched by immersion of the flask in an ice water bath. To passivate the NC surfaces by means of ligand capping, 3 mL of OA was injected 15 – 60 s after Sn injection. Injection before 15 s did not result in NC formation, attributed to the preferential formation of tin oleate in solution. Post-quenching OA injection (i.e., after $\sim$ 60 s) did not effectively

passivate the NC surface and resulted in nanoparticle agglomeration.

S1.2.1 NC Purification

For post-synthesis purification (i.e., washing) in air, the crude SnTe solution was washed with 1:1 $CHCl_3$:acetone v/v and resuspended in nonpolar solvent (e.g., hexane, toluene, $CHCl_3$) for storage in an N_2 glovebox. Any additional washing was performed immediately prior to use with acetone as an antisolvent. Precipitation using alcohols (i.e., methanol, ethanol) as the antisolvent severely damaged the NCs, resulting in their inability to be resuspended. Only two washings in ambient atmosphere were possible, with additional attempts resulting in agglomeration and precipitation of NCs. This behavior was attributed to oxidation. All solvents were purged with N_2 for at least 15 minutes prior to use to remove dissolved oxygen.

For NC purification under inert atmosphere, washing was moved into an N_2 glovebox to maintain the entire synthesis train air free. All solvents employed were stored over activated 3 Å molecular sieves for several days prior to use. Solvents dried in this manner were filtered using 0.22 µm PTFE syringe filters to remove residual sieve dust from solution. It was found that for OA capped NCs, 1:1 $CHCl_3$:acetone v/v produced only limited precipitation, while neat acetone damaged the NCs and they did not effectively resuspend in nonpolar solvents. For OA-capped crude SnTe, washing with 1:1 $CHCl_3$:acetonitrile (MeCN) v/v resulted in the formation of a biphasic liquid with no precipitate. Therefore, the initial precipitation of the crude SnTe was performed using 1.4:1 $CHCl_3$:MeCN to solubilize the OA. Subsequent washings for OA capped NCs, and all washing for those capped in OAm, used 1:1 $CHCl_3$:MeCN as the antisolvent. Toluene or $CHCl_3$ were used as the solvent. Three washes were typically performed on samples used for absorbance spectroscopy.

S1.2.2 NC Oxidation via Processing and Elemental Composition

As explained in the Results section of the main document, NCs were subjected to three different synthesis and processing conditions to identify sources of oxidation. Namely, these conditions were (1) OA-capping of the NCs and purification under ambient atmosphere (atmosphere washed), (2)

OA-capping and washing in an inert atmosphere glovebox (GB washed), and (3) OAm-capping and washing in an inert atmosphere glovebox (OAm capped). A single synthesis was conducted as explained in the preceding paragraphs with minor alterations, but in particular scaled by $\frac{1}{2}$ (e.g., 7 mL OAm, etc.). Immediately prior to OA injection and quenching, a 3 mL aliquot was collected and injected into an N_2-purged bomb flask cooled in an ice water bath. This aliquot represented the OAm capped NC condition. The remaining crude product received an OA injection (scaled for the reduced reaction volume; 0.5 mL) and subsequent quenching. Both the OA and OAm capped crude product were transferred into a N_2 glovebox for subsequent processing.

Once in the glovebox, the OA-capped product was split into two equal fractions, one washed under atmosphere and one under inert atmosphere. While it was desired to wash all samples subjected to different processing in an identical manner (e.g., solvent/anti-solvent ratio, number of washes), this was found to be impossible in practice because of the differing solution qualities encountered.

The OA-capped, atmosphere washed fraction (4 mL) was removed from the glovebox and washed with 7:5 $CHCl_3$/MeCN, at a net ratio of 1:2:1.5 crude SnTe/$CHCl_3$/MeCN. This $CHCl_3$/MeCN solution was not degassed or dried. A slight excess of $CHCl_3$ was necessary to prevent formation of a biphasic mixture. The solution was centrifuged 15 min at 4000 rpm (2700 ×g) and resuspended in 2 mL toluene (not degassed or dried). The sample was then freeze-pump-thawed and returned to the N_2 glovebox for storage. To prepare the sample for elemental analysis, it was subjected to 9 additional washing cycles under inert atmosphere to fully purify the sample. A solution of 1:10:10:10 SnTe-tol/tol/$CHCl_3$/MeCN (all solvents dried and degassed) was centrifuged 5 minutes at 13,000 rpm (11,400 ×g). The subsequent 8 washings consisted of resuspension of the pellet in 50 µL toluene (dry, degassed) and precipitation with 100 µL 1:1 $CHCl_3$/MeCN (dry, degassed). The final pellet was allowed to air dry in the glovebox, was resuspended in 50 µL toluene (dry, degassed), and then drop cast onto a lacy-carbon TEM grid or Si wafer substrate for analysis by TEM/EDS or XPS, respectively (see Sections S1.11 and S1.14).

The OA-capped, GB washed fraction (4 mL) remained in the glovebox and was washed with 7:5 $CHCl_3$/MeCN, at a net ratio of 1:3.5:2.5 crude SnTe/$CHCl_3$/MeCN (all solvents dried and de-

gassed). It was found that significantly more antisolvent was necessary to precipitate the NCs for this processing condition, possibly caused by reduced oxidation and subsequent ligand loss with respect to the atmosphere washed fraction. The solution was centrifuged 30 min at 3200 rpm ($\sim$ 1000 $\times$g) and resuspended in 2 mL toluene. To prepare the sample for elemental analysis, it was subjected to 9 additional washing cycles under inert atmosphere. A solution of 1:5:7.5:7.5 SnTe-tol/tol/$CHCl_3$/MeCN was centrifuged 5 minutes at 13,000 rpm (11,400 $\times$g). The subsequent 8 washings consisted of resuspension of the pellet in 50 µL toluene (dry, degassed) and precipitation with 100 µL 1:1 $CHCl_3$/MeCN (dry, degassed). The final pellet was allowed to air dry in the glovebox, was resuspended in 50 µL toluene (dry, degassed), and then drop cast onto a lacy-carbon TEM grid or Si wafer substrate for analysis by TEM/EDS or XPS, respectively (see Sections S1.11 and S1.14).

The OAm-capped, GB washed fraction (3 mL) was washed with 1:1 $CHCl_3$/MeCN, at a net ratio of 1:1:1 crude SnTe/$CHCl_3$/MeCN (all solvents dried and degassed). The solution was centrifuged 30 min at 3200 rpm ($\sim$ 1000 $\times$g) and resuspended in 1.5 mL toluene. To prepare the sample for elemental analysis, it was subjected to 3 additional washing cycles under inert atmosphere. A solution of 1:10:10:10 SnTe-tol/tol/$CHCl_3$/MeCN was centrifuged 5 minutes at 3200 rpm (690 $\times$g). The subsequent 2 washings consisted of resuspension of the pellet in 100 µL toluene (dry, degassed) and precipitation with 200 µL 1:1 $CHCl_3$/MeCN (dry, degassed). The final pellet was allowed to air dry in the glovebox, was resuspended in 50 µL toluene (dry, degassed), and then drop cast onto a lacy-carbon TEM grid or Si wafer substrate for analysis by TEM/EDS or XPS, respectively (see Sections S1.11 and S1.14). The reduced number of purification cycles with respect to other processing conditions was necessary because of pronounced NC loss and colloidal instability when additional washings were performed. This is attributed to the significantly greater lability of OAm compared to OA. Additional OAm could be added to repassivate the NC surface, but this was counterproductive for sample purification.

S1.3 Reaction Monitoring

Reaction monitoring was conducted to elucidate NC formation mechanisms. Two manners of reaction monitoring were conducted for these studies: by the collection of samples during a typical hot-injection synthesis, with samples collected after each reagent addition (denoted as Sample 1, Sample 2, etc.); and by the collection of aliquots during a single-pot heat-up synthesis (denoted as Aliquot 1, Aliquot 2, etc.). Hot-injection samples consisted of the crude reaction mixture: initially containing OAm and TOPTe (Sample 1), after injection of the tin silylamide ODE mixture (Sample 2), after injection of OA during NC growth (Sample 3), and post-quenching of the reaction (Sample 4). This experimental setup allowed for the identification of reaction products produced upon the addition of a given precursor. It prevented, however, the observation of any reaction kinetics, since each sample represented both an addition (i.e., injection) and removal (i.e., sample withdrawal) of mass from the chemical system. To overcome this shortcoming, a single-pot heat-up synthesis where aliquots were collected was performed. During this synthesis, a mixture of OAm, TOPTe, and tin silylamide was ramped (1800 °C/hr) to 110 °C, held at temperature for 60 sec, injected with OA, and the reaction quenched. Aliquots were collected prior to heating (20 °C, Aliquot 1), at 60 °C (Aliquot 2), at 110 °C (Aliquot 3), and after OA injection and quenching (Aliquot 4).

Because of the poor sensitivity of some nuclei investigated via NMR, particularly ^{125}Te, modifications were made to the precursor ratios. For the hot injection sample synthesis, the tin silylamide and TOPTe loadings were increased 5-fold relative to other precursors. The typical 1:1.75 Sn:Te ratio was maintained. For the single pot aliquot synthesis, tin silylamide and TOPTe loadings were increased 8-fold while maintaining a 1:1.75 Sn:Te ratio. Further, a higher concentration TOPTe solution was used (1.8 M Te), representing a 1:1.2 Te:TOP ratio. Lastly, ODE was eliminated from the synthesis as all previous quantitative NMR spectra of the characteristic ^{1}H peaks at $\delta \sim 5.6$ ppm and $\delta \sim 4.8$ ppm indicated that ODE was not participating in any reactions. Details of the specific syntheses are provided in Table S2

In all cases, regardless of these changes, NC formation still occurred. Significant broadening of the size distribution and an increase in mean particle diameter resulted (Figure S17). More im-

portantly, all significant NMR peaks matched those observed during a 'typical' synthesis (Section S1.2), indicating that the NC formation mechanisms were identical between synthetic methods.

S1.4 Reagent Analyses

S1.4.1 OAm tin silylamide OA (OOA carboxamide)

Tin silylamide, OAm, and OA were reacted and compared to crude SnTe synthesis aliquots to demonstrate the production of N-(cis-9-octadecenyl)-oleamide (oleyloleamide, OOA). OAm (70%, 0.726 g, 2.72 mmol), OA (0.217 g, 0.77 mmol), and tin silylamide (0.320 g, 0.73 mmol) were heated at 110 °C for 10 minutes with stirring in a N_2 glovebox. A color change from strong yellow to weakly yellow (nearly colorless) was observed on heating. The solution composition was chosen to match that of a single pot aliquot synthesis (see Table S2). FTIR absorption was performed on this solution in a NaCl IR cell (Figure S13).

To confirm the presence of OOA, a 1:1:1 solution of tin silylamide:OAm:OA was prepared and analyzed via NMR spectroscopy. First, OAm (98%, 0.156 g, 0.585 mmol) and tin silylamide (0.273 g, 0.621 mmol) were heated at 110 °C for 30 minutes with stirring in a N_2 glovebox. This ensured complete reaction of OAm and tin silylamide to form tin oleylamine (**1**) prior to introduction of OA. Then, OA (0.188 g, 0.666 mmol) was injected and the solution heated at 110 °C for an additional 20 minutes with stirring. Upon cooling, 0.102 g of this solution (0.096 mmol OAm, 0.102 mmol tin silylamide, 0.110 mmol OA) was combined with benzene-d6 (0.451 g, 5.35 mmol) and analyzed for ^{1}H, ^{13}C, and ^{119}Sn (Figure S14).

S1.4.2 1:1:3 OAm tin silylamide OA

SnTe NC syntheses employ an excess of OA with respect to tin silylamide. Therefore, OAm and tin silylamide were reacted with an excess of OA in order to identify potential SnTe NC reaction pathways. First, OAm (98%, 0.121 g, 0.454 mmol) and tin silylamide (0.192 g, 0.437 mmol) were heated at 110 °C for 35 minutes with stirring in a N_2 glovebox. This ensured complete reaction of

OAm and tin silylamide to form **1** prior to introduction of OA. Then, OA (0.452 g, 1.60 mmol) was injected and the solution heated at 110 °C for an additional 30 minutes with stirring. Visible evolution of gas bubbles was observed upon OA injection. 60 seconds after OA injection, a solution aliquot was collected and quenched by injection into a vial kept in a 0 °C cold well. This aliquot was collected in order to identify stable reaction intermediates. For the aliquot, 0.249 g of the solution (0.147 mmol OAm, 0.142 mmol tin silylamide, 0.520 mmol OA) was combined with benzene-d6 (0.487 g, 5.79 mmol). For the completely reacted solution, 0.204 g of the solution (0.121 mmol OAm, 0.116 mmol tin silylamide, 0.426 mmol OA) was combined with benzene-d6 (0.396 g, 4.71 mmol). Both samples were analyzed for ^{1}H, ^{13}C, and ^{119}Sn by solution NMR spectroscopy (Figure S11).

S1.4.3 1:1:1 HMDS OA OAm

HMDS, OA, and OAm were reacted to demonstrate that Sn, not its silylated ligands, is necessary for the formation of OOA. First, HMDS (0.191 g, 1.19 mmol) and OA (0.328 g, 1.16 mmol) were heated at 110 °C for 30 minutes with stirring in a N_2 glovebox. HMDS and OA formed a white, waxy solid upon combining at room temperature, but became clear with visible gas evolution on heating. This ensured complete reaction of HMDS and OA to form a silylated oleate ester prior to introduction of OAm. A small aliquot of the HMDS OA mixture was collected for analysis. Then, OAm (98%, 0.322 g, 1.20 mmol) was injected into the remaining solution and the solution heated at 110 °C for an additional 30 minutes with stirring. Upon cooling, 0.073 g of the HMDS OA OAm solution (0.103 mmol HMDS, 0.101 mmol OA, 0.105 mmol OAm) was combined with benzene-d6 (0.422 g, 5.02 mmol) and analyzed for ^{1}H and ^{13}C (Figures S11, S14). NMR spectroscopic results showed no significant reaction with OAm, suggesting a strong preference by HMDS for the silylation of carboxylates over primary amines in this system.

S1.4.4 1:1 OA OAm

OA and OAm were reacted to investigate the role that tin silylamide played in the formation of OOA. OAm (70%, 0.428 g, 1.60 mmol) and OA (0.445 g, 1.58 mmol) were heated at 110 °C for 53 minutes with stirring in a N_2 glovebox. Upon cooling, 0.096 g of this solution (0.176 mmol OAm, 0.173 mmol OA) was combined with benzene-d6 (0.512 g, 6.13 mmol) and analyzed for ^{1}H and ^{13}C (Figure S14).

S1.4.5 Tin silylamide OAm (Job plot)

OAm and tin silylamide were reacted at various ratios to determine *in situ* precursors formed during SnTe NC syntheses. These data were used to develop a Job plot (Section S2.2; Figures 5); the raw data for which can be found in Table S3. OAm and tin silylamide were combined and heated at 110 °C for 30 minutes with stirring in a N_2 glovebox. Upon cooling, a portion of each respective solution was combined with benzene-d6 and analyzed for ^{119}Sn, ^{1}H, and ^{13}C (Figure S7).

S1.5 Low Temperature TOPTe NMR

Low temperature ^{31}P and ^{125}Te NMR were used to investigate the nature of the TOPTe resonance peak in solution. A solution of 0.0642 g TOPTe (0.0579 g TOP, 0.152 mmol TOP, 0.0063 g Te, 0.049 mmol Te, 0.72 M TOPTe, 1:3.07 Te:TOP), 0.0057 g HMPA (0.032 mmol), and 0.3899 g THF-d8 (4.86 mmol THF, m.p. $\sim$ -108 °C) was prepared and placed in a J. Young tube. HMPA was used as an internal standard. This sample was cooled to temperatures between 20 °C and -90 °C and solution phase ^{31}P NMR spectra collected (Figure S4(a)). 256 scans with a relaxation delay of 1 second was used for each spectra. This data clearly shows the splitting of the TOPTe-TOP resonance peak into its respective TOPTe and TOP peaks as the temperature decreases. Good resolution of both peaks is observed at temperatures below approximately -70 °C. The chemical shifts for TOPTe and TOP are observed at $\sim$ -9.3 and $\sim$ -32.5 ppm, respectively.

Because of the relatively poor sensitivity of ^{125}Te NMR, a solution significantly enriched in Te

was used for low temperature Te NMR spectroscopy. A solution of 0.1538 g TOPTe (0.1203 g TOP, 0.315 mmol TOP, 0.0335 g Te, 0.263, 1.8 M TOPTe, 1:1.20 Te:TOP) and 0.3464 g THF-d8 (4.32 mmol THF, m.p. ~ -108 °C) was prepared and placed in a J. Young tube. This sample was cooled to temperatures between 20 °C and -100 °C and solution phase ^{125}Te NMR spectra collected (Figure S4(b)). At least 256 scans, with a relaxation delay of 1 second was used for each spectra. At this higher concentration, good resolution of the TOPTe doublet is observed at temperatures as high as -50 °C and below. The chemical shifts for TOPTe is observed between ~ -820 and -850 ppm as the temperature decreases from 20 °C to -100 °C, respectively. The well-known shifting of Te peaks with temperature is clearly observable with these data.[2]

S1.6 SnTe NC synthesis using pre-reacted tin silylamide and OAm

OAm and tin silylamide were pre-reacted for a SnTe NC synthesis to confirm the role of the highly-reactive tin oleylamine (**1**). Initially, the OAm and tin silylamide were reacted to produce **1**. 2.643 g OAm (70%, 6.92 mmol) was dried and degassed on a Schlenk line at 120 °C for 70 minutes under vacuum. After which, the solution was placed under N_2 and the temperature was dropped to 100 °C. Once at temperature, 0.1726 g tin silylamide (0.393 mmol) was injected into the OAm, which immediately turned yellow and slowly darkened to orange-brown over the course of about 10 minutes. OAm and tin silylamide were stirred for 1 hr at 100 °C, and then allowed to cool to room temperature.

In a separate 25 mL round bottom flask, 3.4417 g OAm (70%, 9.01 mmol) and 2.6367 g ODE (9.40 mmol) were dried and degassed under vacuum for 1.5 hours at 120 °C. Once dry, 0.490 g TOPTe (0.369 mmol, 0.68 M TOPTe, 1:3.14 Te:TOP) was injected into the OAm/ODE solution. Mass of the TOPTe syringe was measured before and after injection to determine the net amount of Te injected. The solution temperature was then raised to 110 °C and injected with 1.5547 g of the pre-reacted tin silylamide-OAm solution (0.217 mmol tin silylamide, 3.82 mmol OAm). The reaction was allowed to proceed for 60 seconds, and then was quenched to room temperature with an ice water bath. The crude NC solution was transferred to a N_2 purged bomb flask and brought

into an inert-atmosphere glovebox for NC purification and storage.

During a ’typical’ SnTe synthesis (Section S1.2), a solution of tin silylamide and ODE is injected into a degassed OAm and TOPTe solution. To maintain consistent molar ratios, in essence an equal amount of OAm and ODE were exchanged for this synthesis with respect to a ’typical’ synthesis. A comparison of syntheses is presented in Table S2. An absorption spectrum, TEM micrographs, and NC mean diameter results are presented in Figure S15.

S1.7 UV/Vis Absorption Spectroscopy

UV-Vis absorption spectra were taken using a 1 cm pathlength PTFE-pinned visible/NIR quartz cuvette (operating wavelengths: 220 - 3800 nm) or a PTFE-pinned quartz cuvette (operating wavelengths: 170 - 2700 nm) on a Perkin-Elmer Lambda 950 UV/Vis/NIR spectrophotometer. Tetrachloroethylene was used as the NC solvent.

S1.8 FTIR Absorption Spectroscopy

FTIR absorption spectra were collected on a Shimadzu IRPrestige-21 Fourier transform infrared spectrophotometer, typically using a NaCl IR cell with a nominal 0.1 mm path length and averaged over at least 64 scans.

S1.9 Fluorescence Spectroscopy

Photoluminescence spectra were obtained on a home-built fluorometer system consisting of a 450 W Xe arc lamp or a 633 nm 15 mW HeNe laser source coupled to a SpectraPro 150 monochromator system for excitation, and a SpectraPro 300i monochromator equipped with a liquid-N_2 cooled InSb detector for emission detection. A ZnSe lens was used to focus monochromator emission onto the InSb detector active area. The detector output was fed through an EG&G 5209 lock-in amplifier referenced to an optical chopper which was used to chop the excitation between 400 – 900 Hz. All samples were excited at 633 nm. NC emission was investigated from 1550 to 2800 nm (443 to 800

meV, 3571 to 6452 cm^{-1}), which was dictated by the detector cutoff at short wavelengths and the monochromator grating at long wavelengths.

S1.10 Mass Spectrometry

For analysis of tin silylamide oleylamine molecules, samples were prepared in a manner consistent with Section S1.4.5. OAm and tin silylamide were reacted at ratios of 1:1 and 1:4 tin silylamide:OAm and heated at 110 °C for 30 minutes with stirring in a N_2 glovebox. Further, prior to heating, an aliquot of the 1:4 tin silylamide:OAm sample was collected for comparison of the sample pre- and post-heating. In total, three samples were analyzed: (1) 1:1 Sn:OAm, (2) 1:4 Sn:OAm, and (3) 1:4 Sn:OAm (unheated).

Initial attempts to collect acceptable mass spectrometry (MS) spectra used atmospheric pressure chemical ionization (APCI) or electrospray ionization (ESI) techniques. Samples were diluted in dry dichloromethane and loaded into a gas tight syringe prepared in a glovebox for transport to the MS. Samples were directly injected into the MS ionization chamber (i.e., no chromatography was performed). MS spectra collected using these two techniques showed no isotope patterns consistent with tin. The reason for this is unclear, given that the samples were maintained air-free and sample insertion was performed under N_2. However, we could only conclude that the samples were degrading via oxidation or hydrolysis in some manner.

Ultimately, direct insertion probe (DIP) MS was used (EI, 70 eV). For DIP MS, a sample was prepared in a glovebox and loaded into a quartz cup. If necessary, that sample was diluted in dry pentane (1:3 v/v sample:pentane) to better control sample loading into the quartz cup, as very little material is needed so as not to overload the MS detector. The pentane was allow to evaporate, and then the quartz cup was sealed with camphene and placed in a sealed scintillation vial for transport to the MS to ensure the sample remained air and water free throughout. The sample was loaded into the sample holder and subjected to light vacuum ($\sim 10^{-4}$ Torr) in the MS antechamber to remove the camphene and expose the sample. The sample was then fully inserted into the MS, and the probe was heated from 50 °C to 350 °C at a rate of 80 °C min^{-1}. Mass spectra were averaged over the entire

collected signal, and background subtracted to remove baseline peaks. Peaks were normalized to the strongest intensity peak, and signals with $< 1\%$ relative intensity were also removed. Additional results discussion is provided in Section S2.3.

S1.11 Transmission Electron Microscopy & Energy Dispersive X-ray Spectroscopy

Transmission electron microscopy (TEM) micrographs were taken on a FEI TECNAI F-20 field emission microscope at an accelerating voltage of 200 kV using samples suspended in toluene and drop cast on lacey carbon grids (Ted Pella). Energy-dispersive x-ray (EDX) spectroscopy was performed using a TEM-integrated EDAX Octane T Si drift detector (SDD) spectrometer. Ensemble NC size statistics were collected using ImageJ (National Institutes of Health).[3] For size histogram development, the Sturge's method for optimal bin width determination was used.

S1.12 Nuclear Magnetic Resonance Spectroscopy

Proton (^{1}H), phosphorus (^{31}P), tin (^{119}Sn), and silicon (^{29}Si) NMR spectra were recorded at ambient temperature on an Avance 500 (500 MHz) spectrometer. Tellurium (^{125}Te) NMR spectra were recorded at ambient temperature on an Avance 400 (400 MHz) spectrometer. Chemical shift (δ) was recorded in ppm. An external calibration standard was employed using a coaxial insert NMR tube or sealed capillary, which provided a standard for comparison of relative intensities and to relate concentrations without the possibility of the standard reacting with the species being studied.

For quantitative NMR (qNMR) of P, Sn, and Te, external capillary standards' concentrations were validated against compounds of known concentration prior to use. Validation for H and C was performed by means of a mass balance, whereby the known moles of all material added to the system was compared to the total measured moles. For all nuclei, results of this validation found errors in concentration of less than 10 per cent. NMR spectroscopy acquisition parameters and reference compounds are presented in Table S4.

^{1}H NMR spectra were referenced to the solvent residual benzene peak at $\delta^{^1H}$ = 7.16 ppm,[4] and quantitative ^{1}H NMR were measured relative to an external Me_4Sn sealed capillary standard. Similarly, ^{13}C NMR spectra were referenced to the solvent residual benzene peak at $\delta^{^{13}C}$ = 127.83 ppm,[5] and quantitative ^{13}C NMR were measured relative to an external Me_4Sn sealed capillary standard. ^{31}P NMR spectra were quantified and referenced to an external HMPA sealed capillary standard at $\delta^{^{31}P} = 25.0$ ppm, whose chemical shift was itself referenced to 85% H_3PO_4 H_2O at $\delta^{^{31}P}$ = 0.0 ppm. ^{119}Sn NMR spectra were quantified and referenced to an external Me_4Sn sealed capillary standard at $\delta^{^{119}Sn} = 0.0$ ppm. ^{125}Te NMR spectra were quantified and referenced to an external Ph_2Te_2 sealed capillary standard at $\delta^{^{125}Te} = 420.8$ ppm.[6] ^{29}Si NMR spectra were referenced to an external HMDSO sealed capillary standard at $\delta^{^{29}Si} = 7.22$ ppm.[7]

Heteronuclear multiple bond correlation (HMBC) was employed to identify couplings between ^{1}H$-$^{13}C, ^{1}H$-$^{119}Sn, and ^{1}H$-$^{29}Si nuclei. Suppression of bond coherence is achieved when the preparation and refocusing delay (D2) equals $\frac{1}{2\,J_{X-^1H}}$, which was utilized in ^{1}H$-$^{13}C HMBC to suppress single bond H-C correlations (e.g., $^1J_{^1H-^{13}C}$). Tin compounds, however, have a significantly broader range of coupling constant values ($^nJ_{^1H-^{119}Sn}$, n = 1 - 6).[8] Therefore, it is not feasible to selectively suppress single bond correlations, only correlations which match a given frequency. Further, an extended long-range coupling delay (D6) was used to try and capture the most long distance correlations possible. This can lead to noisier spectra and weakening of shorter-distance couplings though relaxation damping, but can promote the appearance of smaller couplings.[8,9] These modifications were utilized for both ^{1}H$-$^{119}Sn and ^{1}H$-$^{29}Si HMBC spectroscopy, and are detailed in Table S4.

S1.13 X-ray Diffraction Crystallography

X-ray diffraction (XRD) was performed on a Philips PANalytical MPD PW3020 diffractometer using Cu Kα (λ = 1.540 56 Å) radiation. Phase identification was made in X'Pert Data Viewer software by comparison to Joint Committee on Powder Diffraction Standards (JCPDS) reference files. Alternatively, data was collected using a Rigaku XtaLAB Synergy-S Dualflex diffractometer

equipped with a HyPix-6000HE Hybrid Photon Counting area detector and using a PhotonJet Cu Kα x-ray source.

S1.14 X-ray Photoelectron Spectroscopy

X-ray photoelectron spectra were collected on a Kratos AXIS Ultra DLD spectrophotometer with an incidence angle of 60° and a take-off angle of 90°. The hybrid lens spot size was 0.7 mm × 0.4 mm (0.28 mm^2). Unless otherwise noted, samples were prepared on cleaned Si substrates in an N_2 glovebox, and carried in a sealed container to the instrument. Charge neutralization was used to minimize charge accumulation of the sample during data collection, although on occasion the neutralizer was unnecessary with well purified, sufficiently thin samples.

For sample substrates, appropriately sized pieces of p++ doped Si wafer (B dopant) were sonicated sequentially in nanopure water, acetone, and isopropanol for 5 minutes, 5 minutes, and 10 minutes, respectively. Cleaned substrates were dried in air overnight under a watchglass to minimize dust contamination, and then pumped into an inert atmosphere glovebox for storage and subsequent sample preparation.

Analyses of XPS spectra were performed using CasaXPS software (Casa Software Ltd), and peak assignments (e.g., SnTe, Te^0) were made based on the comparison of the measured binding energy with literature values.[10] Spectral baselines were fit with Shirly (C, O, Si) or Tougaard (Sn, Te, Cd, Se, S) background algorithms and subtracted. Peaks were fit with pseudo-Voigt curves (70% Lorentzian), using a minimum number of peaks to provide an adequate fit of the spectrum. All peaks for a given high-resolution spectrum were constrained to have a constant full width half max (FWHM). The only exception was for the fitting of Si 2p SiO_2 peaks, which had a notably larger FWHM with respect to other measured Si peaks (Figure 2). Spin-orbit splitting peak spacing and area ratios were also constraints, with values taken from literature. Spin-orbit spacings used were: Si 2p = 0.63 eV, Sn 3d = 8.4 eV, Te 3d = 10.4 eV , Cd 3d = 6.8 eV, Se 3d = 0.86 eV, and S 2p = 1.16 eV. Constrained peak area ratios used were for p (1:2) and d (2:3) subshells. Relative sensitivity factors (RSFs) for each element were calculated within CasaXPS based upon theoretical Scofield

values, modified by acquisition parameters (e.g., source-analyzer angle). A thin layer escape depth correction with a thickness estimation parameter ('MFP exp.') of 3 was used, and a 30° source analyzer angle. Calculated RSFs include: C 1s 1.0; O 1s 2.93; Si 2p 1.49; Sn 3d 41.75; Te 3d 51.58; Cd 3d 20.22; Se 3d 2.18; and S 2p 1.68.

After peak fitting within CasaXPS, a total integrated peak area normalized by the element's RSF was used to determine the elemental composition for a sample. Spectra were collected from at least two locations on a sample film (rarely more), and the results between both locations averaged to determine the overall sample composition.

Mathematically, the elemental ratio between two elements is calculated as

$$\frac{n_1}{n_2} = \frac{I_1/S_1}{I_2/S_2}$$

where $\frac{n_1}{n_2}$ is the relative ratio of the two elements, I_i is the measured peak area, and S_i is the RSF. This can be extrapolated to all measured elements in a sample, and an element's composition (C_x) within a sample is calculated as

$$C_x = \frac{n_x}{\Sigma n_i} = \frac{I_x/S_x}{\Sigma I_i/S_i}$$

.

S2 Results and Discussion

S2.1 Surface Characterization by XPS

XPS involves the irradiation of a sample material with monochromatic soft x-ray photons, which cause the ejection of electrons of a characteristic energy. These x-ray photons are able to penetrate to a depth of between approximately $1 - 10\,\mu\text{m}$ depending on the material being analyzed. However, sampling depth (or information depth) is determined by the emitted electrons, which can travel only a much shorter distance before interacting with the parent sample material and recombining. The mean distance the electrons can travel before inelastically scattering is referred to as the inelastic

mean free path (IMFP, λ). Similar to the x-ray penetration depth, the IMFP is material dependent. However, it is generally on the order of 10 nm or less. Thus, only electrons very close to the surface are able to escape the material inelastically and be detected.[11,12]

Disregarding elastic scattering effects, the sampling depth (Σ) of a material from which 95% of the detected signal originates is approximated as

$$\Sigma = \lambda \cos(\alpha) \ln(20)$$

where α is the photoelectron emission angle with respect to the surface normal. Given this, electrons may be ejected from the material surface equal to a depth of approximately 3λ (given $\alpha = 0$ for an orthogonal orientation). The sampling depth distribution of ejected electrons is exponential. Thus, approximately 65% of electrons are ejected from a depth of $\leq \lambda$, 20% from between λ - 2λ, 10% from between 2λ - 3λ, and 5% from depths $\geq 3\lambda$.[12,13] SnTe is reported to have an IMFP of 2.7 nm when irradiated with Al Kα x-rays, resulting in a Σ = 8.1 nm.[14]

In contrast, EDS operates on the principle of detection of characteristic x-rays. These x-rays, emitted from a sample after electron absorption, allow for the detection and identification of constituent elements within a sample. The interaction volume of the electrons with the sample material is defined by the Kanaya-Okayama equation, which states that

$$R = 0.0276 \frac{A \cdot E_0^{\frac{5}{3}}}{Z^{\frac{8}{9}} \rho}$$

where R is the electron depth penetration (µm), A is the atomic weight (g/mol), E_0 is the electron beam energy (keV), Z is the atomic number, and ρ the material density $\left(\frac{g}{cm^3}\right)$. Given this equation, for elements ranging from C (Z = 6) to U (Z = 92) and having beam energies characteristic of TEM (~ 100 - 300 keV), one finds R ~ 1 µm.[15]

Therefore, EDS is an effective supplement to XPS. While the sample depth of SnTe NCs from XPS is on the order of the nanoparticles' full diameter, the signal is weighted towards just the surface (~ 2.7 nm). Therefore, much of the signal will be collected from the SnTe NC oxide shell. EDS,

with a sampling depth on the order of 1 μm, will analyze the full volume of the NC and can therefore be taken as a more robust measure of the overall composition. Using these two complementary measures of elemental composition, one can begin to understand differences in the NC surface composition versus overall particle composition.

As validation of concept, elemental composition data were collected and averaged for XPS (n = 2) and EDS (n = 10) spectra on a sample of CdS NCs (Figure S18). Previous work has shown that CdS NCs are approximately stoichiometric, with a slight Cd excess arising from having a Cd terminated surface.[16] Comparison of these data show nearly identical composition regardless of technique, with a slight excess of Cd as expected. This lends confidence in the SnTe elemental composition data presented in Table S1. Regardless, the resulting data show a significant inconsistency in the elemental composition of SnTe measured by XPS versus EDS. It may be partially explained by the presence of a Sn-deficient (non-stoichiometric) core and a Sn-rich oxide shell. Further, there may be significant error in the peak fitting used for determination of the elemental composition by EDS. The Cd and S peaks are well separated in the EDS spectrum, facilitating quantitation. However, Sn and Te show significant overlap that may lead to errors in peak fitting and resultant quantitation (Figure S18(g, h)).

S2.2 Job plot

The method of continuous variations (MCV), in its simplest form, can be used to understand the equilibrium composition between two reacting species [A] and [B] that form a binary complex [AB] (i.e., [A] + [B] $\rightleftharpoons$ [AB]). MCV maintains a constant sum concentration of [A] and [B] while varying their individual relative proportions (e.g., [A] + [B] = constant). By adjusting the concentrations of the two reagents, information about the resulting product can be gleaned visually via a simple plot. The plotted abscissa is the mole fraction of one constituent. For example, $\chi_A = \frac{[A]}{[A]+[B]}$. The ordinate axis data type varies, but could potentially be any measure that correlates linearly with the concentration of AB. Most commonly employed is UV-vis absorption data, but other data that have been successfully used include: conductivity, permittivity, circular dichroism,

and NMR spectroscopy. A plot of the concentration of a reagent (P_A) with respect to χ_A should appear as an approximate bell-shaped curve, and is known as a Job plot. From such a Job plot, one can infer the stoichiometric ratio of two constituents in the product molecule [AB]. For example, a curve showing a peak at χ_A = 0.5 indicates a 1:1 stoichiometric ratio for [AB] (i.e., [A_nB_n]). A curve with a peak at χ_A = 0.33 would indicate a 1:2 A/B ratio (i.e., [A_nB_{2n}]). With sufficiently detailed data, more complex interpretation can be made. For example, several curves can be plotted to identify multiple product species resulting from the reaction between [A] and [B]. Further, fitting of the curve with one of several models can be used to extract the equilibrium constant for the reaction.[17] Details on the syntheses of samples used for preparation of the Job plot in this work are found in Section S1.4.5, and the raw data collected for plot development can be found in Table S3.

S2.3 Mass Spectrometry Discussion

Mass spectrometry spectra were collected via direct insertion probe (DIP) MS per Section S1.10. In total, three tin silylamide:OAm samples were prepared and analyzed: (1) 1:1 Sn:OAm, (2) 1:4 Sn:OAm, and (3) 1:4 Sn:OAm (unheated). The selected tin silylamide:OAm ratios were chosen to be enriched in Compound **1** (1:1 Sn:OAm, 100%) and Compound **2** (1:4 Sn:OAm, ~ 20%) as per our MCV analysis (Table 3)

Below approximately m/z = 300, all tin silylamide:OAm sample spectra were dominated by signal originating from the OAm molecular ion [$OAm^{+\bullet}$] and OAm fragments [$M{-}nCH_2^{+\bullet}$], and thus focus was placed on the spectral region m/z > 300. However, no notable spectral differences are observed between samples (Figure S8).

Difference spectra were prepared for (**a**) 1:4 Sn:OAm - 1:4 Sn:OAm (unheated) spectra and (**b**) 1:4 Sn:OAm - 1:1 Sn:OAm samples (Figure S8(g)). Such spectra were prepared by subtracting the relative intensity of one spectrum at a given m/z (Y) from that of another (X), e.g., X – Y. The difference spectra for **a** (1:4 Sn:OAm - 1:4 Sn:OAm (unheated)) was intended to highlight molecules or features which formed upon sample heating, while difference spectra **b** (1:4 Sn:OAm

- 1:1 Sn:OAm) was intended to highlight features present in the 1:4 solution that were not in the 1:1 solution (e.g., compound **2**). No significant species were able to be identified in this manner.

We attribute this lack of observable mass fragments in the data to the high reactivity of the Sn species, and likely fragmentation of the molecules during ionization. Spectra were also collected using a reduced ion current (EI 20 eV), as we hypothesized this might minimize fragmentation and allow observation of the molecular ions (data not shown). Again, no notable spectral difference was observed aside from a significant reduction in signal intensity (i.e., counts).

Known molecular ions include tin silylamide (m/z 440), dimerized tin silylamide (m/z 880), and OAm (m/z 267). Other potential molecules include **1** (m/z 545) and the tin silylamine·OAm complex (m/z 707).

S3 Tables

Table S 1. Elemental [Sn]:[Te] and [Cd]:[S] ratios obtained via EDS and XPS on SnTe and CdS nanocrystals, respectively. SnTe nanocrystals were subjected to alternate synthesis and purification conditions. Calculated standard deviations are displayed in grey.

	**Sn:Te (mol/mol)**		**TEM Diameter** (nm)
	XPS	EDS	
SnTe: Washed in atmosphere	2.20 ± 0.25 : 1	1.03 ± 0.17 : 1	7.85 ± 1.36
SnTe: Washed in glovebox	1.81 ± 0.18 : 1	1 : 1.22 ± 0.16	6.89 ± 0.93
SnTe: Washed in glovebox, no OA	1.24 ± 0.13 : 1	1 : 1.08 ± 0.09	7.73 ± 1.06
CdS	1.24 ± 0.01 : 1	1.22 ± 0.41 : 1	5.30 ± 0.64

Table S 2. SnTe NC synthesis parameters.

**Precursor**	**One-Pot Heat Up Synthesis with Aliquots (typical)**			**One-Pot Heat Up Synthesis with Aliquots (excess OA)**			**Hot Injection Synthesis with Sample Collection**		
	mass (g)	mmol	molar ratios	mass (g)	mmol	molar ratios	mass (g)	mmol	molar ratios
tin silylamide	0.4780	1.09	1 : 2.04 Sn:Te	0.2769	0.630	1 : 1.56 Sn:Te	0.0832	0.189	1 : 1.75 Sn:Te
Te	0.2825	2.21	1 : 1.22 Te:TOP	0.1252	0.982	1 : 1.20 Te:TOP	0.0422	0.331	1 : 2.88 Te:TOP
TOP	1.003	2.71	1 : 2.49 Sn:TOP	0.4368	1.18	1 : 1.87 Sn:TOP	0.3532	0.953	1 : 5.04 Sn:TOP
OAm	3.755	14.0	1 : 12.9 Sn:OAm	2.298	8.59	1 : 13.6 Sn:OAm	1.073	4.01	1 : 21.2 Sn:OAm
ODE	-	-	-	-	-	-	0.5098	2.02	1 : 10.7 Sn:ODE
OA	0.6151	2.18	1 : 2.00 Sn:OA	1.588	5.62	1 : 8.92 Sn:OA	0.3634	1.29	1 : 6.80 Sn:OA
**Precursor**	**Pre-reacted OAm & tin silylamide**			**Typical Synthesis (Literature Method)**					
	mass (g)	mmol	molar ratios	mass (g)	mmol	molar ratios			
tin silylamide	0.0953	0.217	1 : 1.70 Sn:Te	0.1770	0.403	1 : 1.76 Sn:Te			
Te	0.0471	0.369	1 : 3.24 Te:TOP	0.0906	0.710	1 : 3.05 Te:TOP			
TOP	0.4429	1.20	1 : 5.51 Sn:TOP	0.8023	2.16	1 : 5.37 Sn:TOP			
OAm	4.901	18.3	1 : 84 Sn:OAm	11.38	42.6	1 : 106 Sn:OAm			
ODE	2.6367	10.4	1 : 48.1 Sn:ODE	4.611	18.3	1 : 45.3 Sn:ODE			
OA	-	-	-	2.661	9.42	1 : 23.4 Sn:OA			

Table S 3. Method of continuous variation (Job plot) raw data. Compound **1**, δ ~ 675 ppm. Compound **2**, δ ~ 435 ppm.

χ_{Sn}	**δ = 675 ppm** (mol/L)	**δ = 435 ppm** (mol/L)	**tin silylamide**		**OAm**	
			g	mmol	g	mmol
1	0	0	1.25	2.85	0	0
0.910	0.204	0	1.14	2.59	0.069	0.257
0.684	0.729	0	0.856	1.95	0.240	0.899
0.499	1.421	0	0.625	1.42	0.381	1.43
0.333	0.865	0.082	0.416	0.947	0.508	1.90
0.244	0.557	0.116	0.305	0.693	0.576	2.15
0.195	0.442	0.111	0.243	0.553	0.614	2.29
0.091	0.212	0.046	0.114	0.259	0.692	2.59
0.045	0.106	0.021	0.056	0.127	0.728	2.72
0.021	0.030	0.029	0.026	0.059	0.746	2.79

Table S 4. NMR spectroscopy acquisition parameters and reference standards. Units are displayed in parentheses, and Bruker parameter codes are given in square brackets (as applicable).

**qNMR Method**	**Number of scans** [NS]	**Relaxation delay** (s) [D1]	**Reference capillary compound**‡	**Validation reference**‡	**Error**
^{1}H	16	60	Me_4Sn	mass balance	-4.71%
^{13}C	320	60	Me_4Sn	mass balance	5.95%
^{31}P	64	20	HMPA	Ph_3PO	-5.52%
^{119}Sn	512	3	Me_4Sn	tin silylamide	5.44%
^{125}Te	256	15	Ph_2Te_2	TOPTe	2.46%
$^{29}Si^{\dagger}$	128	10	HMDSO	-	-

**2D NMR Method**‡	**Number of scans** [NS]	**Number of experiments, f1** [TD1]	**FID size, f2** [TD2]	**Relaxation delay** (s) [D1]	**Preparation & refocusing delay** (μs) [D2]	**Long range coupling delay** (s) [D6]	**Gradient recovery delay** [D16]	**Pulse gradients** (g1 : g2 : g3)
$^{1}H-^{13}C$ HMBC	4	1536	16384	1	3450	0.1	200	50 : 30 : 40.1
$^{1}H-^{119}Sn$ HMBC	64	32	32768	2	25.8	0.5	200	50 : 45.65 : 40
$^{1}H-^{29}Si$ HMBC	32	32	16384	2	2	0.5	200	50 : 25.9 : 40
$^{1}H-^{13}C$ HSQC	4	1536	16384	1.5	1725	-	150	80 : 20.1 : 11

† qNMR ^{29}Si spectra were not collected. Because of the long T1 relaxation times for Si nuclei, an extended relaxation delay was used to increase peak intensities.

‡ Me_4Sn: Tetramethyl tin; HMPA: Hexamethylphosphoramide; Ph_2Te_2: Diphenyl ditelluride; HMDSO: Hexamethyldisiloxane; Ph_3PO: Triphenylphosphine oxide; tin silylamide: Bis(bis(trimethylsilyl)amino) tin(II); TOPTe: Trioctylphosphine telluride; HMBC: Heteronuclear multiple bond correlation; HSQC: Heteronuclear single quantum coherence.

S4 Figures

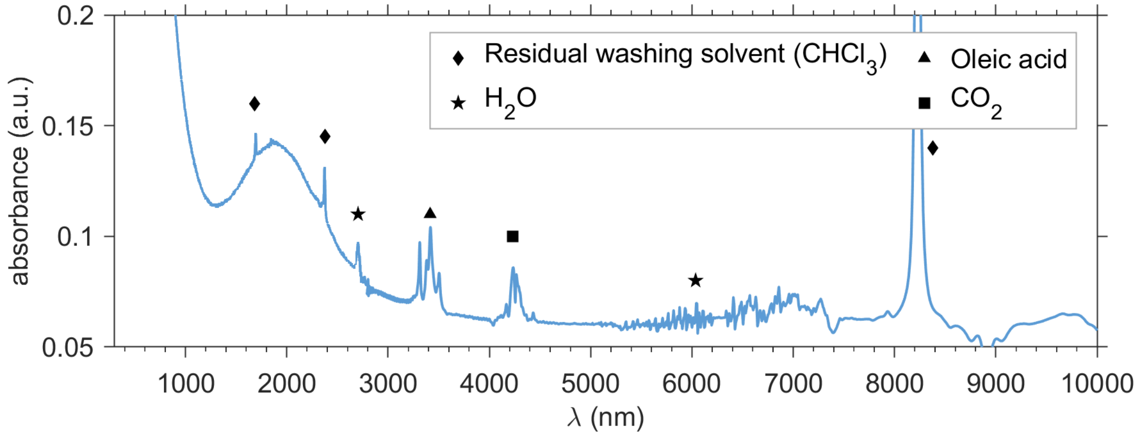

Figure S 1. A typical glovebox washed SnTe NC spectrum.

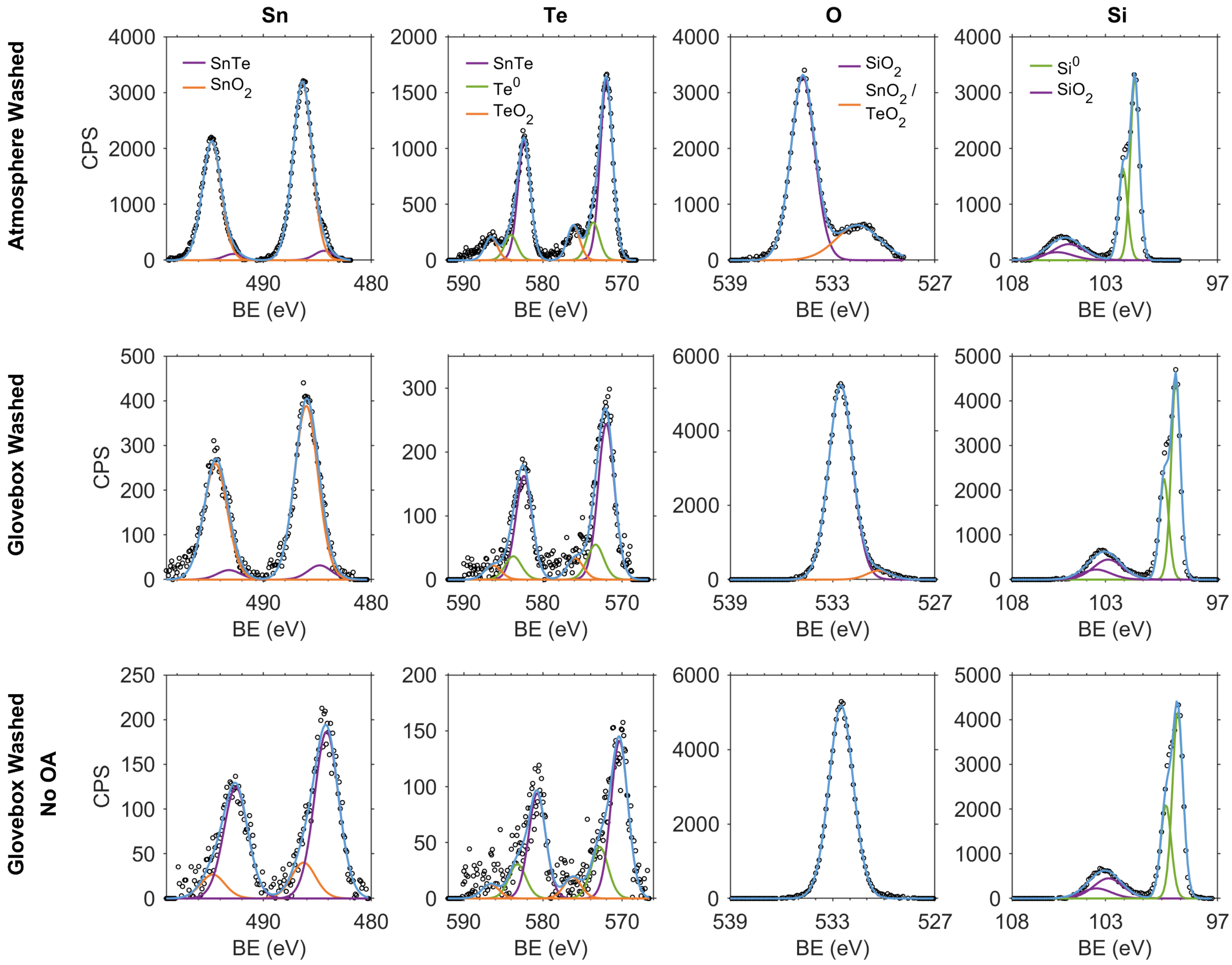

Figure S 2. Sn, Te, O, and Si XPS spectra for NCs subjected to each of the three processing conditions.

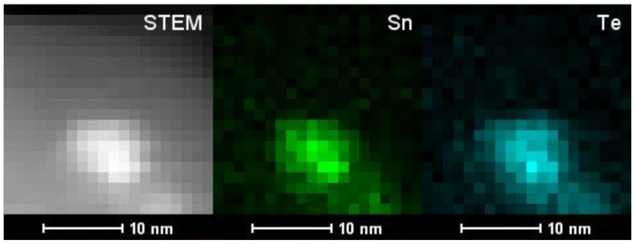

Figure S 3. Typical SnTe NC EDX map. Collected from an OA-capped sample processed by washing in an inert atmosphere.

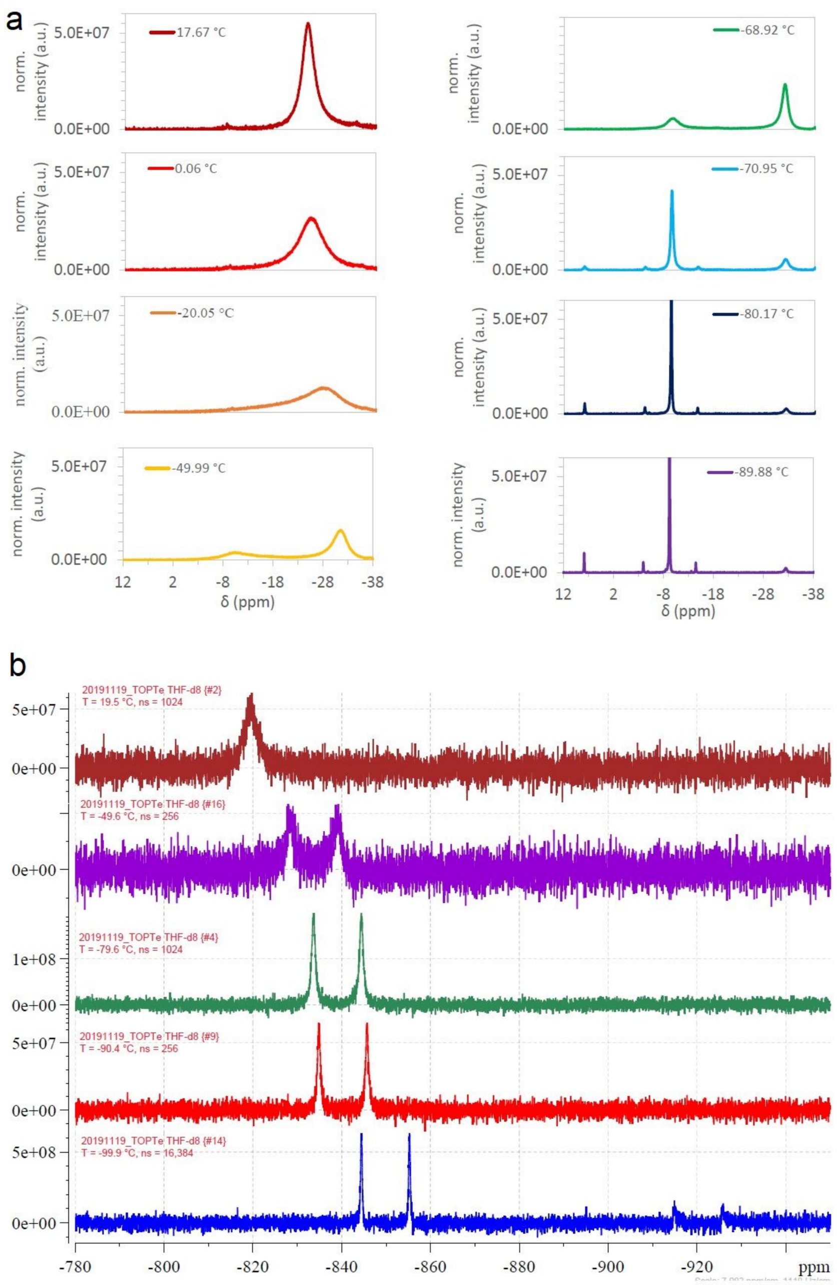

Figure S 4. Variable temperature solution (a) ^{31}P and (b) ^{125}Te NMR spectra of TOPTe in THF-d8.

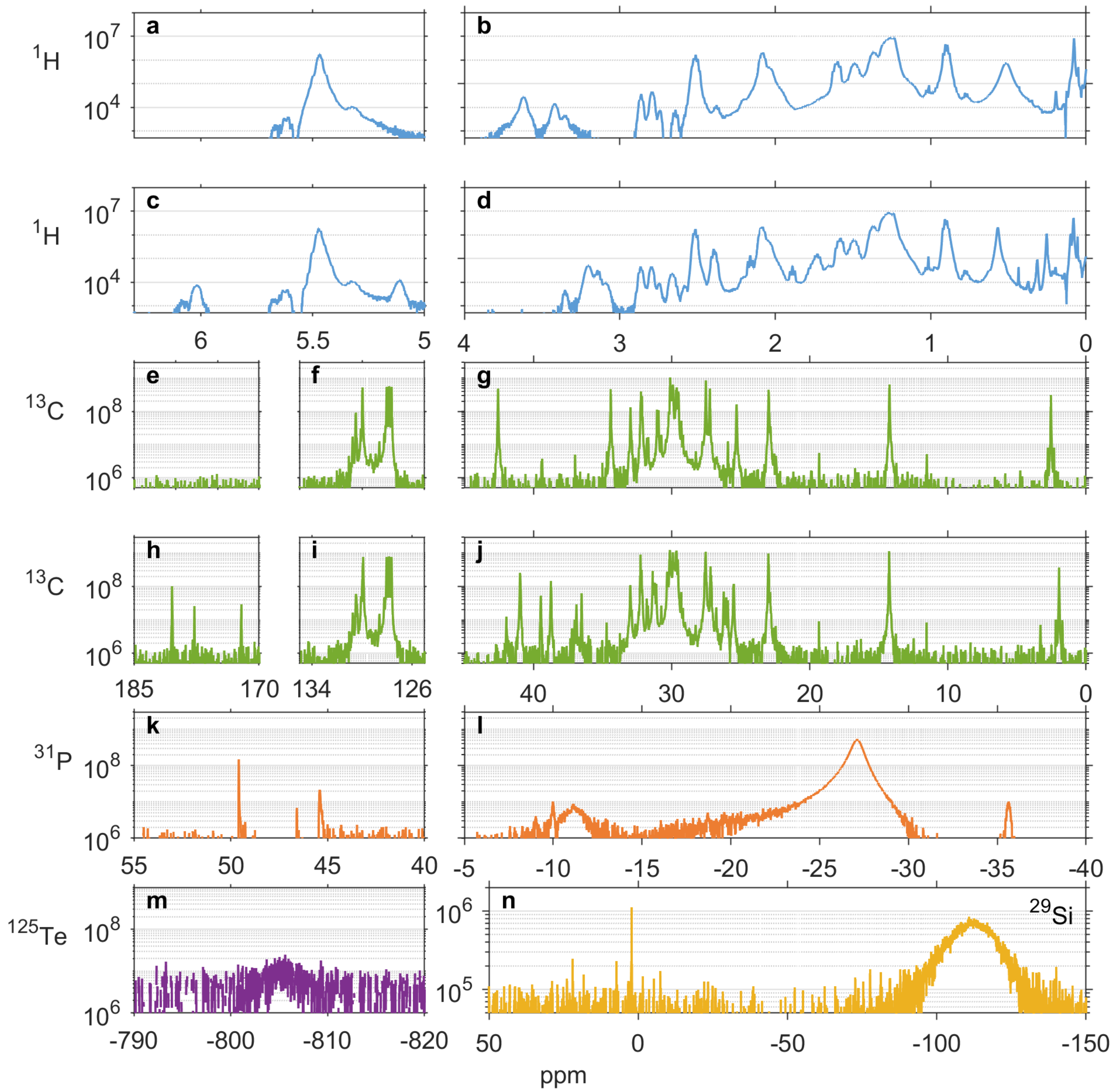

Figure S 5. Crude SnTe reaction product, solution NMR spectra of (a, b) pre-OA injection ^{1}H (Aliquot 3), (c, d) post-OA injection ^{1}H (Aliquot 4), (e - g) pre-OA injection ^{13}C (Aliquot 1), (h - j) post-OA injection ^{13}C (Aliquot 4), (k, l) ^{31}P (Sample 4), (m) ^{125}Te (Aliquot 1), and (n) ^{29}Si (Aliquot 4). Because the data is largely dominated by signal originating from OAm and OA, the spectra are presented in a semi-log format to enhance the visibility of many of the smaller product peaks.

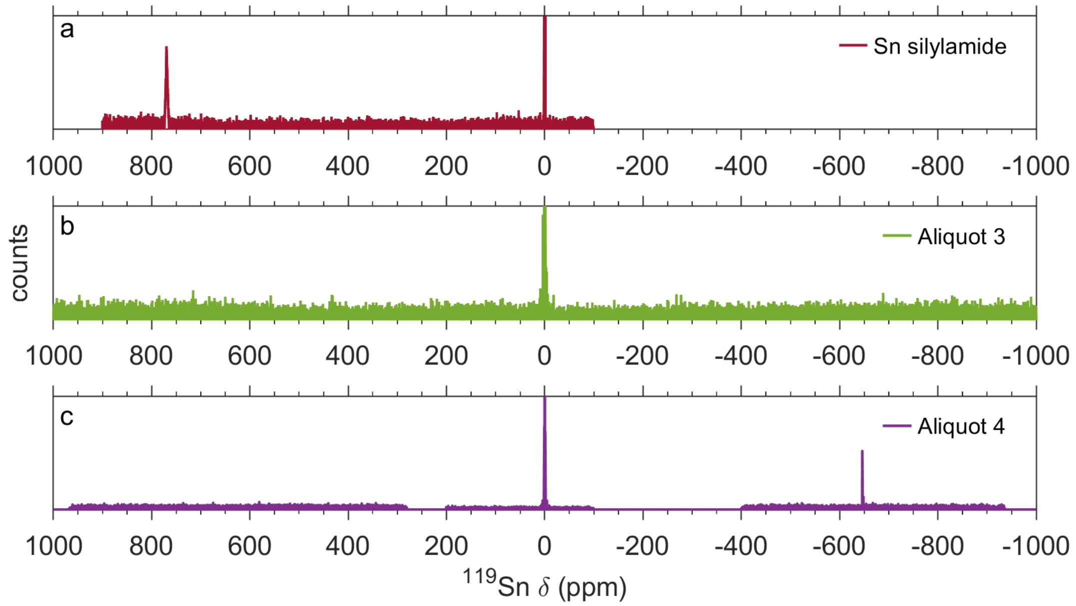

Figure S 6. ^{119}Sn spectra of (a) tin silylamide and synthesis aliquots (b) before and (c) after OA injection. Spectra are referenced to an external Me_4Sn capillary standard.

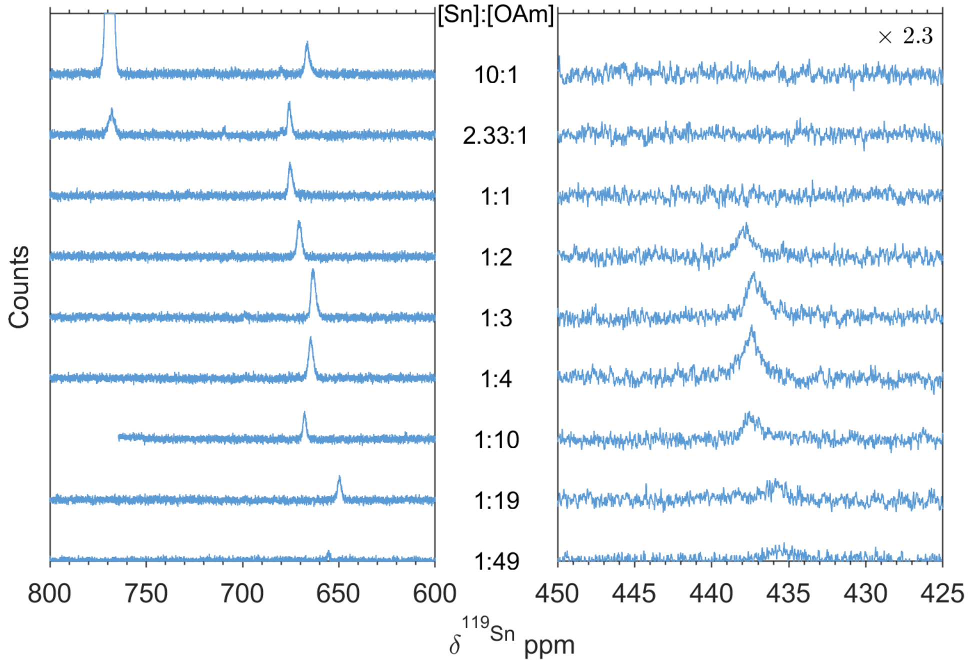

Figure S 7. ^{119}Sn NMR spectra used for the method of continuous variation and the resulting Job plot (Figure 5). Molecular species with chemical shifts at ∼ 775 ppm, 675 ppm, and 435 ppm are identified as tin silylamide, **1**, and **2**, respectively. The shifting of the **1** peak ($\delta \sim 675$ ppm) is consistent with changes in sample viscosity. Samples having a greater proportion of oleylamine or [sample]:[deuterated solvent] ratio show an upfield shift. Details on sample preparation and data analysis are provided in Sections S1.4.5 and S2.2.

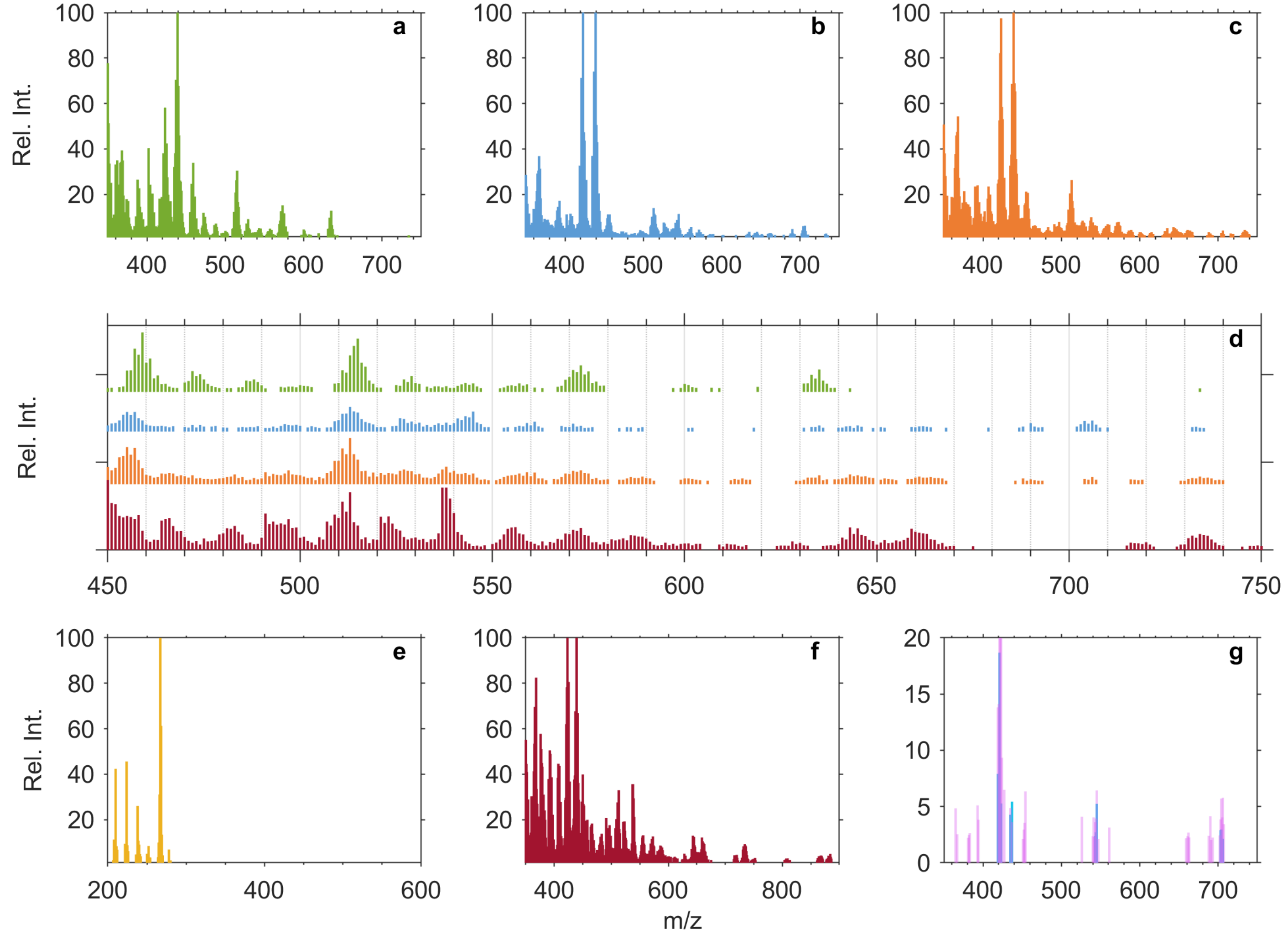

Figure S 8. Mass spectrometry spectra collected via direct insertion probe (DIP) MS per Section S1.10, and normalized at m/z = 440 (tin silylamide molecular ion, $[M^{+\bullet}]$). Mass spectra were collected for mixtures of tin silylamide and OAm reacted at (a) 1:1 Sn:OAm, (b) 1:4 Sn:OAm ratio, and (c) unheated 1:4 Sn:OAm ratios. Panel (d) shows a comparison of (green) 1:1 Sn:OAm, (blue) 1:4 Sn:OAm, (orange) 1:4 Sn:OAm (unheated), and (red) tin silylamide mass spectra. Precursor spectra are shown for (e) OAm and (f) tin silylamide. Panel (g) shows difference spectra for (light blue) 1:4 Sn:OAm - 1:4 Sn:OAm (unheated), and (pink) 1:4 Sn:OAm - 1:1 Sn:OAm. Additional discussion is provided in Section S2.3.

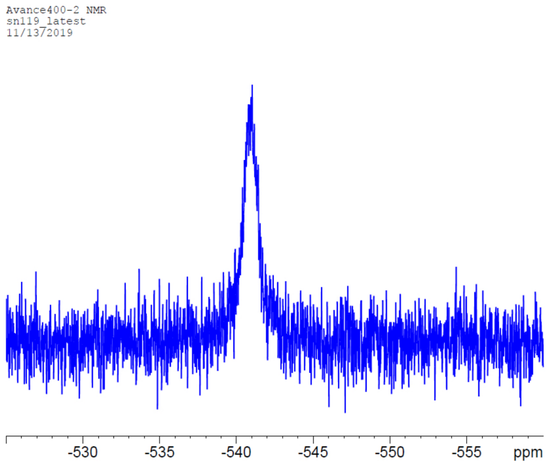

Figure S 9. Sn(II) oleate ^{119}Sn NMR spectrum.

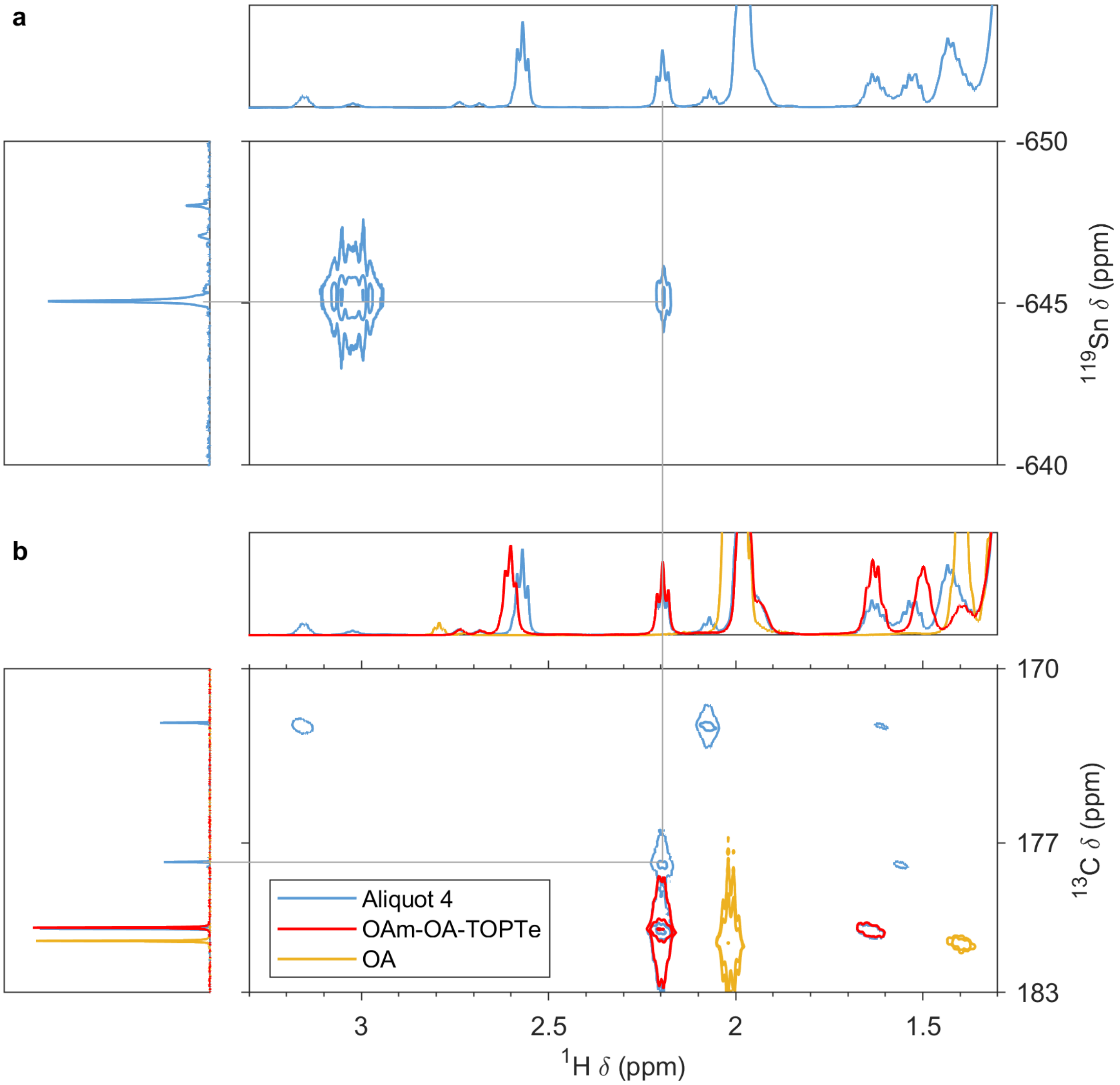

Figure S 10. HMBC spectra of a synthesis aliquot after OA injection (Aliquot 4). (a) ^{1}H-^{119}Sn HMBC. (b) ^{1}H-^{13}C HMBC. Panel (b) compares HMBC spectra of Aliquot 4; OA; and a room-temperature mixture of OAm, OA, and TOPTe to aid in identification of signals associated with Sn-containing molecules. Note that the shifting of the OA ^{13}C carbonyl peak from 180.9 ppm to 180.4 ppm for both the Sample 4 and OAm-OA-TOPTe mixtures is caused by H-bonding interaction between OA and OAm.[18] Additional detail of the alkyl-Sn ^{1}H-^{13}C HMBC carboxylate region is provided in Figure S19.

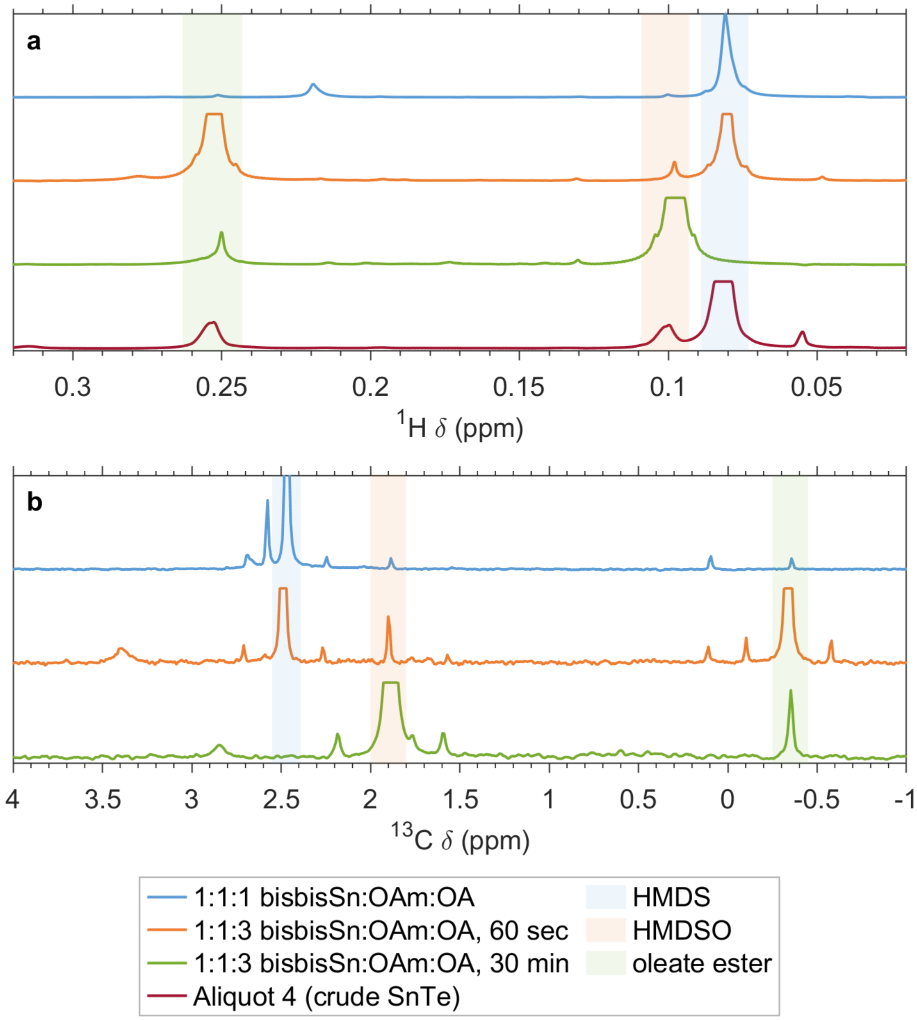

Figure S 11. Solution (a) ^{1}H and (b) ^{13}C NMR spectra of solutions of 1:1:1 bisbisSn:OAm:OA (blue), 1:1:3 bisbisSn:OAm:OA (60 second aliquot, orange), and 1:1:3 bisbisSn:OAm:OA (green). Large peaks are truncated to enhance the visibility of smaller product peaks. In equimolar ratio, the dominant silylated endproduct is HMDS (brown line). With an excess of OA, however, a large portion of trimethylsilyl oleate ester forms at early reaction times (light grey line). As the reaction proceeds, this oleate ester is consumed in the formation of HMDSO (dark grey line). On comparison with a post-OA injection crude SnTe sample (aliquot A4; 1:2:13:2 bisbisSn:TOPTe:OAm:OA), the formation of HMDS, oleate ester, and HMDSO are all readily identified (black line).

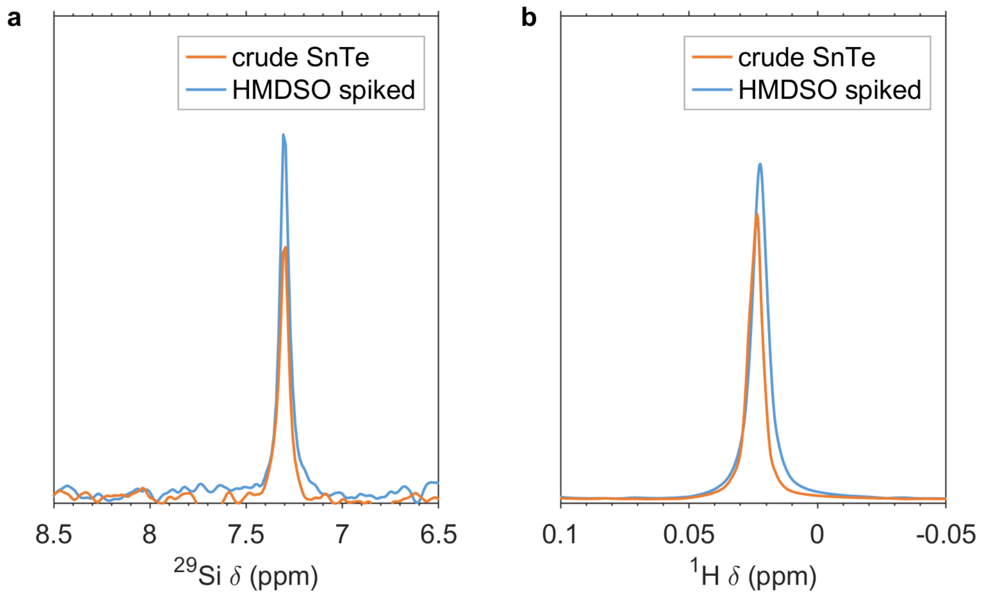

Figure S 12. ^{1}H spectra from a crude SnTe reaction solution (excess OA) that was spiked with HMDSO.

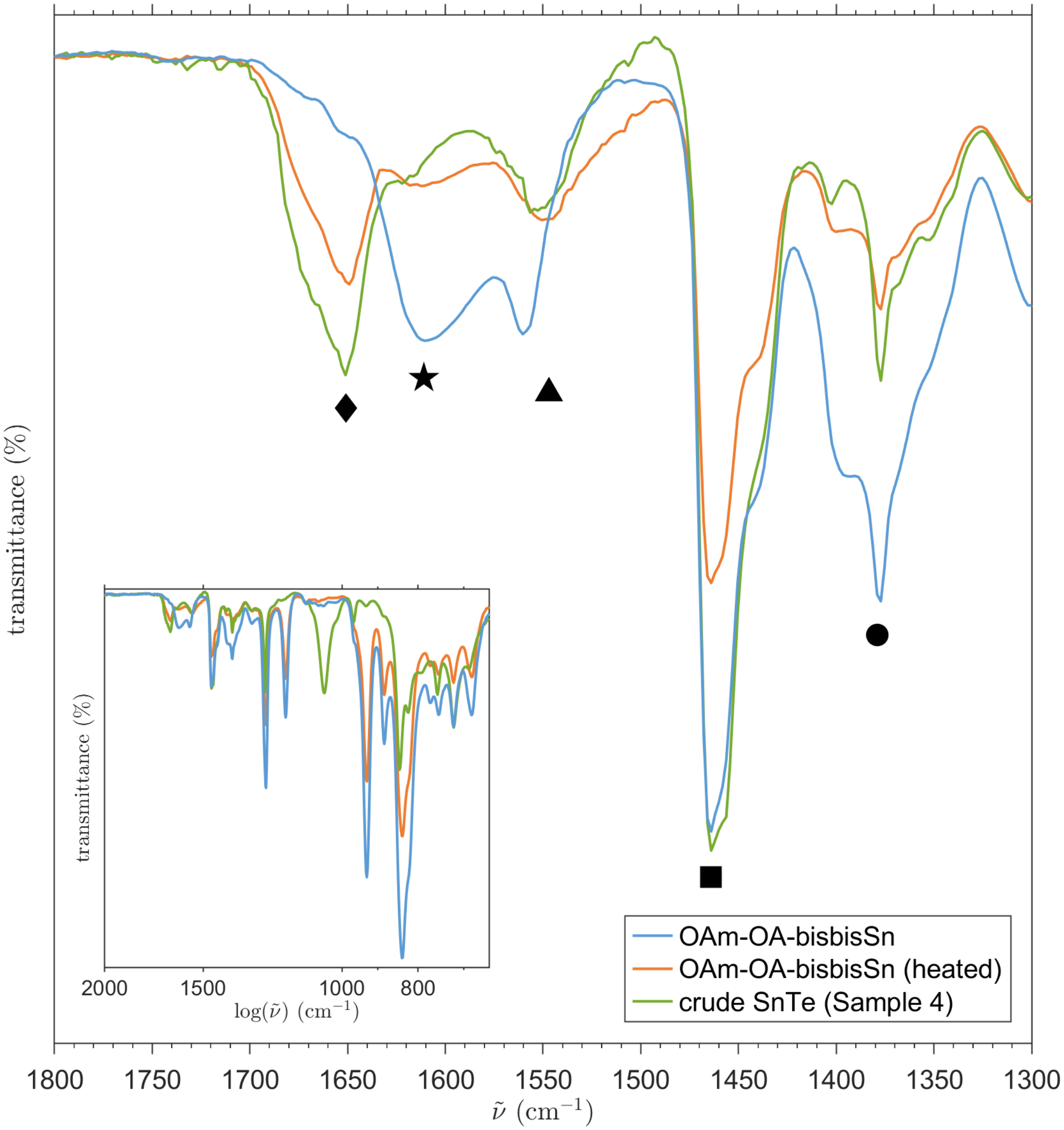

Figure S 13. FTIR spectra of a crude SnTe reaction solution (excess OA) compared to a mixture of OAm, OA, and tin silylamide. The ratios of OAm, OA, and tin silylamide in the mixture approximate those found in a typical NC synthesis. The inset shows additional detail of the FTIR absorption spectrum (fingerprint region). ♦: ν C=O (amide stretch); ★: δ_s NH_2 (scissor/bend); ▲: N-H bend (2° amine); ■: ρ C-H (bend/rock); ●: CH_3 bend.

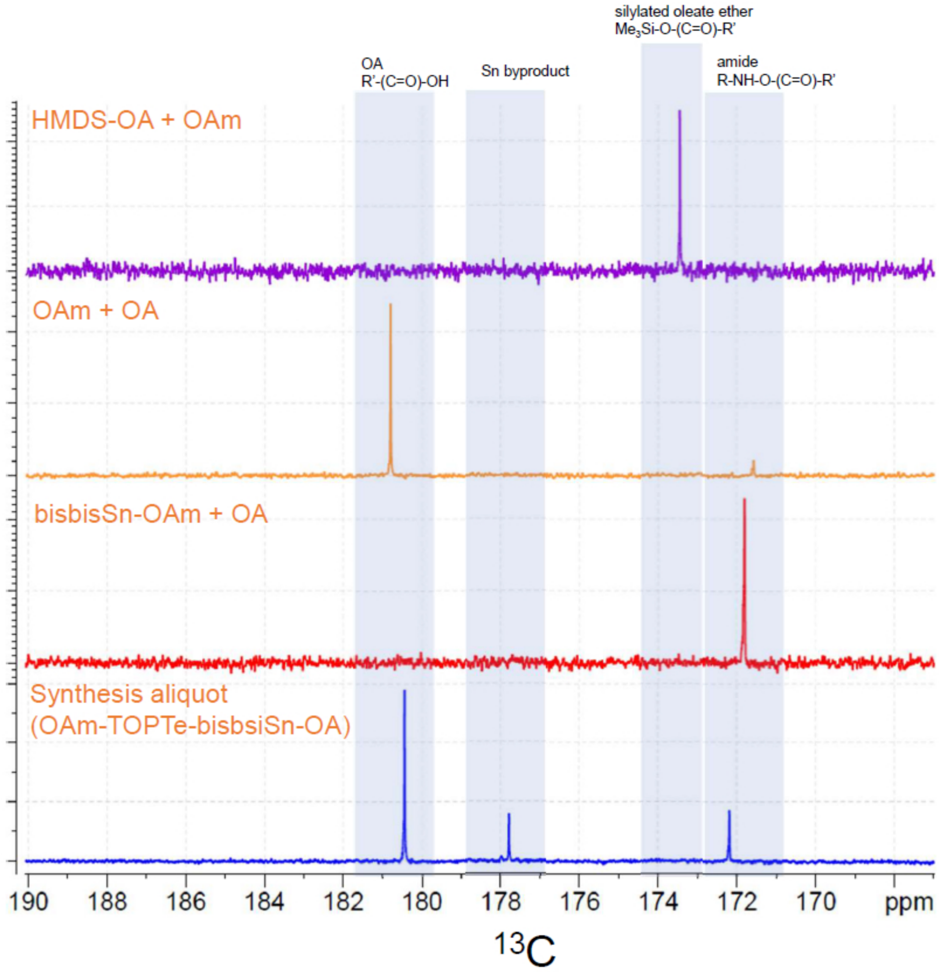

Figure S 14. Carboxylate region ^{13}C NMR spectra for reactions of (1) 1:1:1 HMDS:OA:OAm; (2) 1:1 OA:OAm; (3) 1:1:1 tin silylamide:OA:OAm, and (4) a 'typical' post-OA injection crude SnTe reaction mixture. Little production of oleyloleamide (OOA) occurs from the reaction of OAm and OA, and none when in the presence of HMDS. However, complete conversion of OA to OOA is observed when reacting tin silylamide, OAm, and OA.

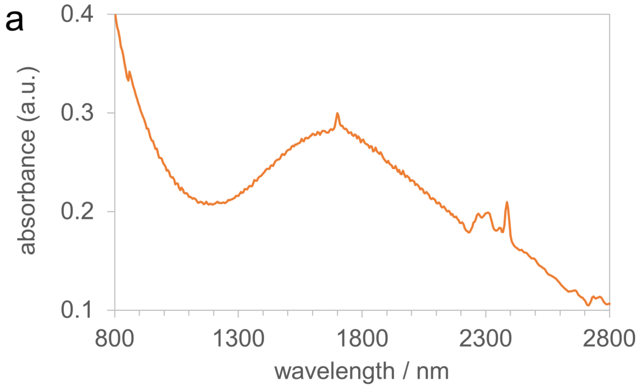

b

Synthesis Ratio Sn:Te	TEM diameter (nm)
‘Typical’ synthesis, OAm capped NCs	7.73 ± 1.0
Pre-reacted bisbisSn + OAm synthesis	6.28 ± 0.82

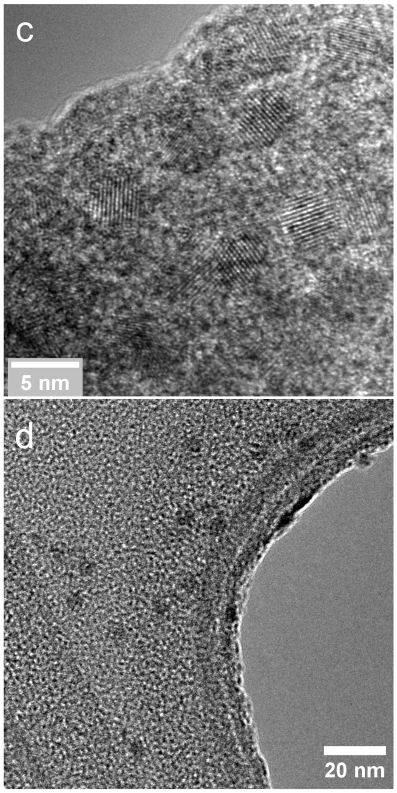

Figure S 15. (a) Absorption spectra, (b) mean diameter comparison, and (c, d) TEM micrographs of SnTe NCs made using an initially reacted tin silylamide and OAm precursor.

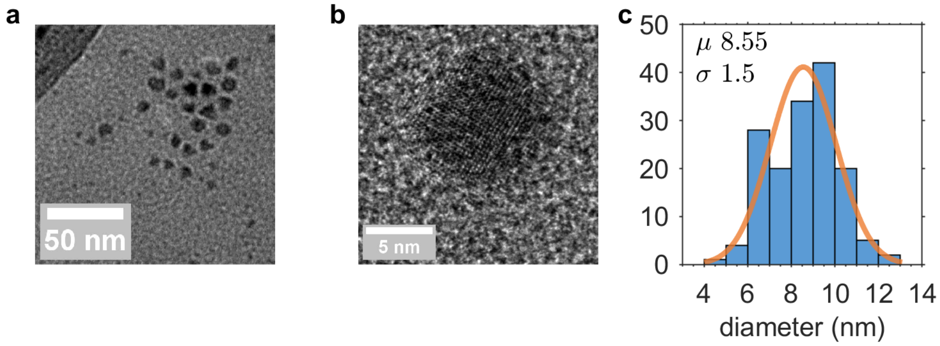

Figure S 16. (a, b) TEM micrographs and (c) size histogram of SnTe NCs synthesized using 98% purity OAm. Sample shows mean NC diameter within the range of that found for NCs synthesized using 70% purity OAm (e.g., $7.2 \pm 0.8 – 8.9 \pm 1.2$ nm).

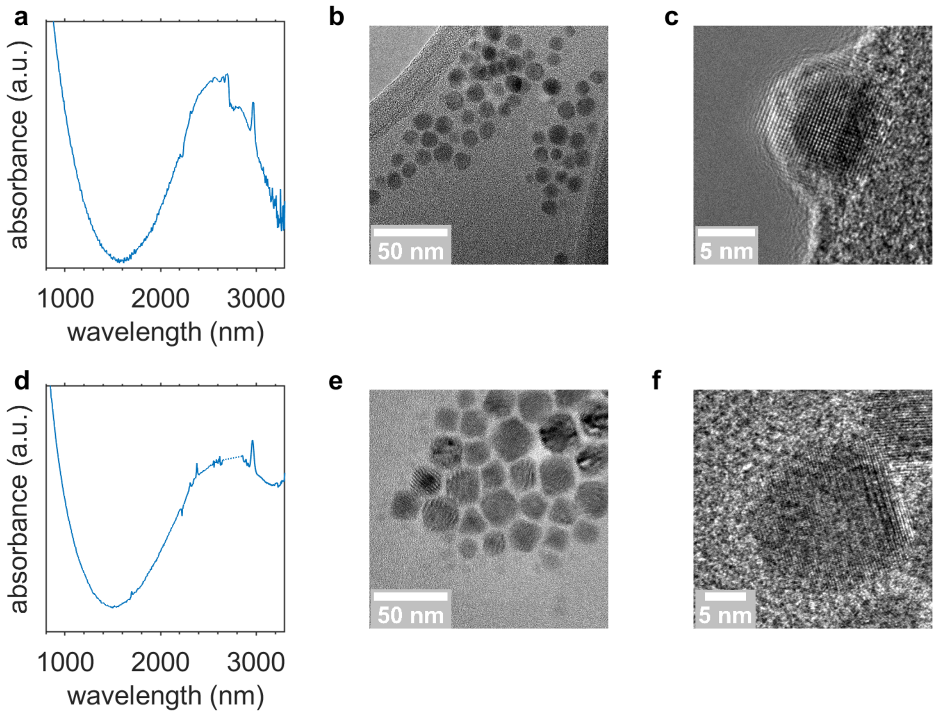

Figure S 17. Typical absorption spectra and TEM for NCs produced using the heat-up synthesis. (a - c) pre-and (d - f) post-OA injection and quenching. The dotted portion of the post-OA injection sample's absorption spectrum indicates the location where absorption by the quartz cuvette was removed. The pre-OA injection sample's absorption was collected in a visible/IR quartz cuvette.

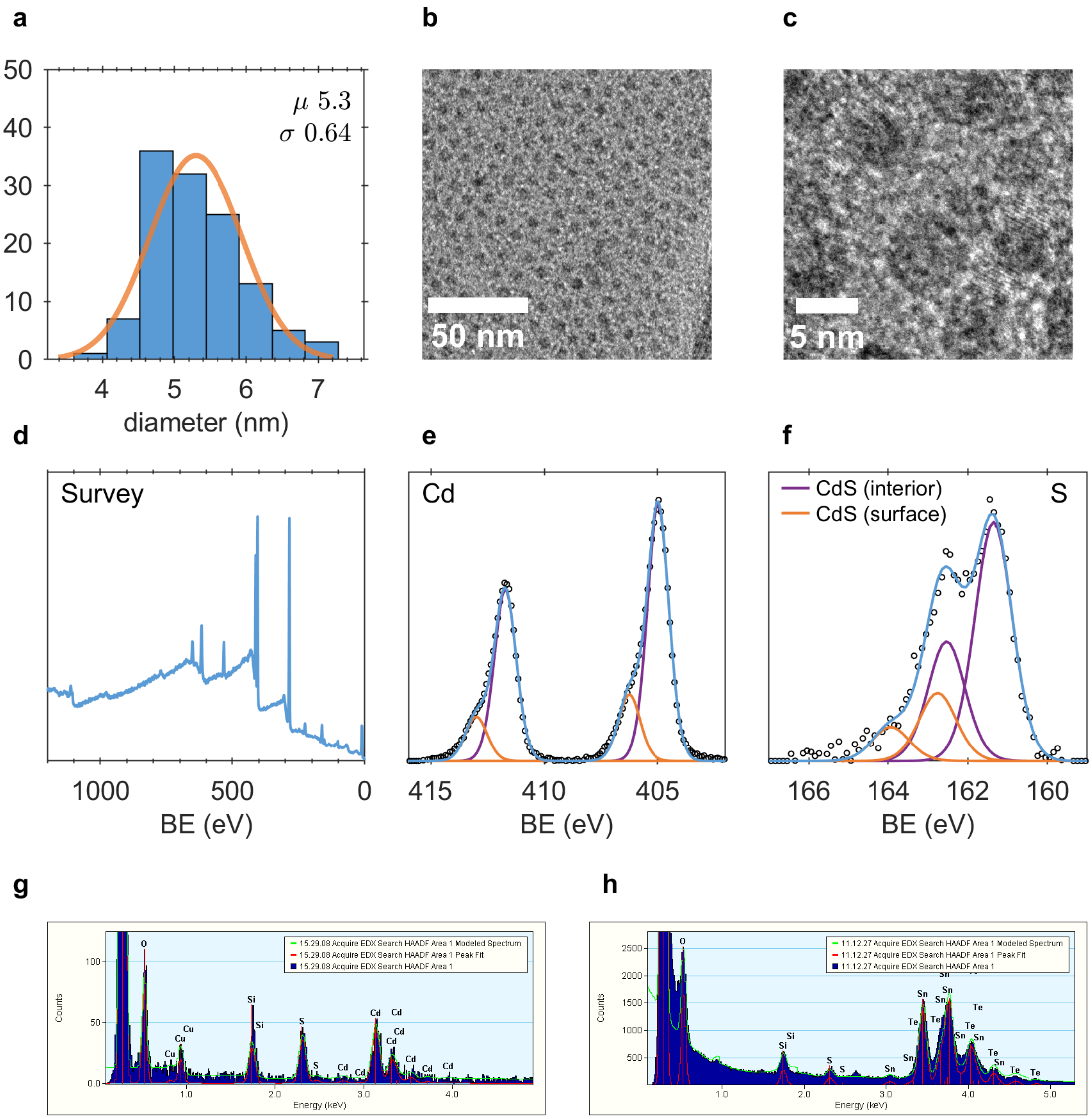

Figure S 18. CdS NC elemental composition data used for validation of SnTe composition results. (a) Nanocrystal size histogram, (b, c) TEM micrographs, (d - f) XPS survey and high-resolution spectra, and examples of (g) a CdS EDS spectrum and (h) a SnTe EDS spectrum. The Cd and S EDS peaks are well separated, while Sn and Te suffer from significant overlap.

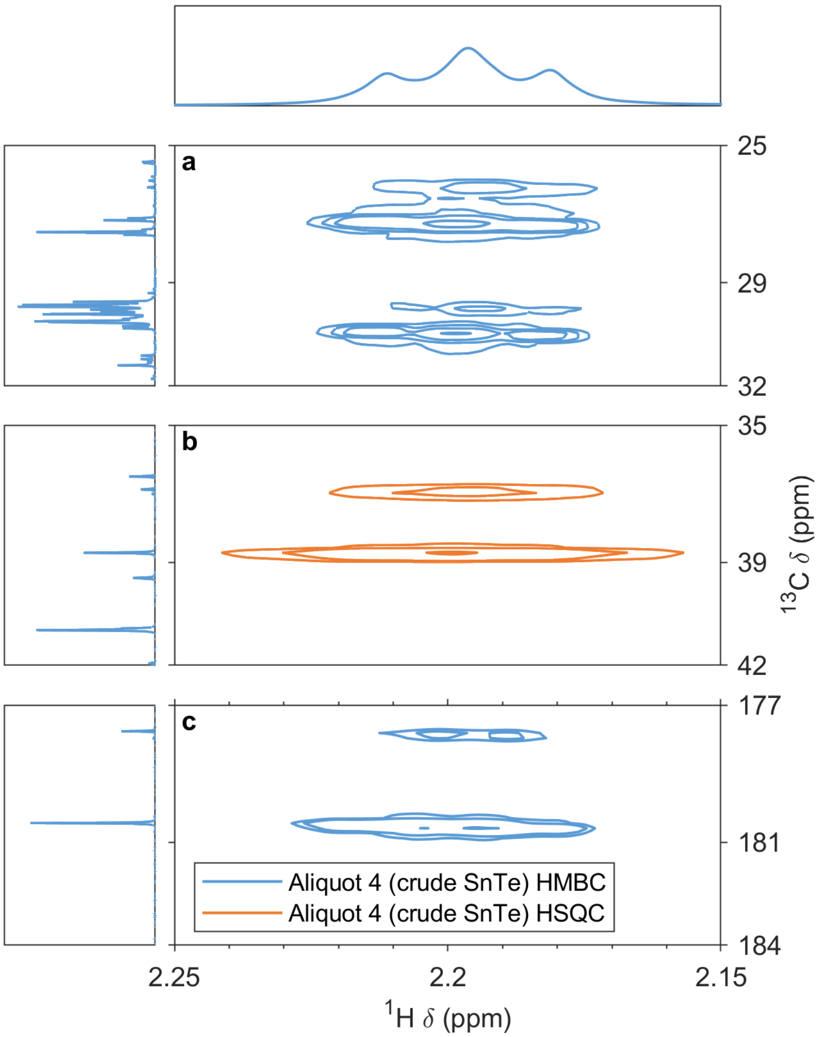

Figure S 19. Post-OA injection crude SnTe solution (Aliquot 4) (a, c) ^{1}H$-$^{13}C HMBC and (b) ^{1}H$-$^{13}C HSQC NMR spectra. These data show that the ^{1}H triplet observed at δ ~ 2.2 ppm consists of two overlapping features attributable to OA's carboxylate-adjacent methylene (i.e., $-CH_2^*-COOH$) and the unidentified alkyl-Sn species. Using both HMBC and HSQC, these nearly coincident crosspeaks are observed consistently. This suggests a similarity in their molecular structures.

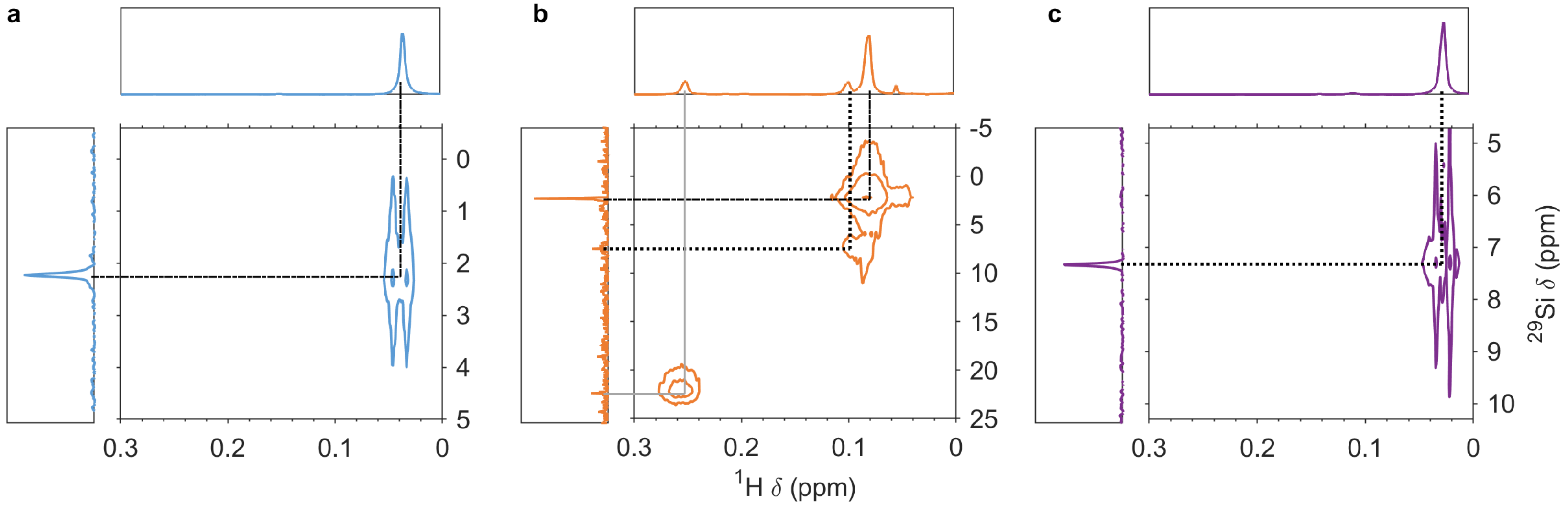

Figure S 20. ^{1}H-^{29}Si HMBC spectra collected for crude SnTe reaction solutions. Spectra represent synthesis where (a) no OA was added, (b) OA was a limiting reagent (i.e., [Sn] > [OA]), and (c) OA was in excess. Note that some shifting is apparent in the ^{1}H NMR spectra, which is attributable to differing sample viscosity.

References

[1] M. V. Kovalenko, W. Heiss, E. V. Shevchenko, J. S. Lee, H. Schwinghammer, A. P. Alivisatos, and D. V. Talapin, "SnTe Nanocrystals: A New Example of Narrow-Gap Semiconductor Quantum Dots," *J. Am. Chem. Soc.*, vol. 129, no. 37, pp. 11 354–11 355, 2007.

[2] C. Brevard and P. Granger, *Handbook of High Resolution Multinuclear NMR*. John Wiley & Sons, 1981.

[3] W. Rasband, "ImageJ," 2022. [Online]. Available: https://imagej.nih.gov/ij/

[4] G. R. Fulmer, A. J. Miller, N. H. Sherden, H. E. Gottlieb, A. Nudelman, B. M. Stoltz, J. E. Bercaw, and K. I. Goldberg, "NMR Chemical Shifts of Trace Impurities: Common Laboratory Solvents, Organics, and Gases in Deuterated Solvents Relevant to the Organometallic Chemist," *Organometallics*, vol. 29, no. 9, pp. 2176–2179, 2010.

[5] "SDBSWeb." [Online]. Available: https://sdbs.db.aist.go.jp

[6] M. Bortoli, M. D. Tiezza, C. Muraro, G. Saielli, and L. Orian, "The 125Te Chemical Shift of Diphenyl Ditelluride: Chasing Conformers Over a Flat Energy Surface," *Molecules*, vol. 24, no. 7, 2019.

[7] H. C. Marsmann, "NMR Spectroscopy, 29Si," in *Encycl. Spectrosc. Spectrom.*, 3rd ed., J. C. Lindon, G. E. Tranter, and D. W. Koppenaal, Eds. Oxford: Academic Press, 2017, pp. 284–293.

[8] J. C. Martins, M. Biesemans, and R. Willem, *Tin NMR Based Methodologies and Their Use in Structural Tin Chemistry*, 2000, vol. 36, no. 4.

[9] F. F. Kayser, M. Biesemans, M. Gielen, and R. Willem, "Demonstrating Small 1H-119Sn Couplings by 1D and 2D Proton-Detected Heteronuclear Multiple-Bond-Correlation Spectroscopy," *J. Magn. Reson. - Ser. A*, vol. 102, no. 2, pp. 249 – 252, 1993.

[10] NIST, "NIST X-ray Photoelectron Spectroscopy Database," 2000. [Online]. Available: https://srdata.nist.gov/

[11] J. F. Moulder and J. Chastain, *Handbook of X-ray Photoelectron Spectroscopy: A Reference Book of Standard Spectra for Identification and Interpretation of XPS Data*. Physical Electronics Division, Perkin-Elmer Corporation, 1992.

[12] F. A. Stevie and C. L. Donley, "Introduction to X-Ray Photoelectron Spectroscopy," *J. Vac. Sci. Technol. A*, vol. 38, no. 6, p. 063204, 2020.

[13] C. J. Powell, "Practical Guide for Inelastic Mean Free Paths, Effective Attenuation Lengths, Mean Escape Depths, and Information Depths in X-Ray Photoelectron Spectroscopy," *J. Vac. Sci. Technol. A*, vol. 38, no. 2, p. 023209, 2020.

[14] H. Shinotsuka, S. Tanuma, C. J. Powell, and D. R. Penn, "Calculations of Electron Inelastic Mean Free Paths. XII. Data for 42 Inorganic Compounds over the 50 eV to 200 keV Range with the Full Penn Algorithm," *Surf. Interface Anal.*, vol. 51, no. 4, pp. 427–457, 2019.

[15] K. Kanaya and S. Okayama, "Penetration and Energy-Loss Theory of Electrons in Solid Targets," *J. Phys. D. Appl. Phys.*, vol. 5, no. 1, pp. 43–58, 1972.

[16] H. H.-Y. Wei, C. M. Evans, B. D. Swartz, A. J. Neukirch, J. Young, O. V. Prezhdo, and T. D. Krauss, "Colloidal Semiconductor Quantum Dots with Tunable Surface Composition," *Nano Lett.*, vol. 12, no. 9, pp. 4465–71, 2012.

[17] J. S. Renny, L. L. Tomasevich, E. H. Tallmadge, and D. B. Collum, "Method of Continuous Variations: Applications of Job Plots to the Study of Molecular Associations in Organometallic Chemistry," *Angew. Chemie - Int. Ed.*, vol. 52, no. 46, pp. 11 998–12 013, 2013.

[18] E. Hassanabadi, M. Latifi, A. F. Gualdrón-Reyes, S. Masi, S. J. Yoon, M. Poyatos, B. Julián-López, and I. Mora-Seró, "Ligand & Band Gap Engineering: Tailoring the Protocol Synthesis

for Achieving High-Quality CsPbI3 Quantum Dots," *Nanoscale*, vol. 12, no. 26, pp. 14 194–14 203, 2020.
